# Openness to experience predicts eye movement behavior during scene viewing

**DOI:** 10.3758/s13414-024-02937-z

**Published:** 2024-08-12

**Authors:** Nicholas J. Wyche, Mark Edwards, Stephanie C. Goodhew

**Affiliations:** School of Medicine and Psychology, Australian National University, Canberra, Australia

**Keywords:** Visual attention, Attentional breadth, Eye movements, Working memory, Personality

## Abstract

Individuals’ *abilities* to perform goal-directed spatial deployments of attention are distinguishable from their broader *preferences* for how they use spatial attention when circumstances do not compel a specific deployment strategy. Although these preferences are likely to play a major role in how we interact with the visual world during daily life, they remain relatively understudied. This exploratory study investigated two key questions about these preferences: firstly, are individuals consistent in their preferences for how they deploy their spatial attention when making shifts of attention versus adopting an attentional breadth? Secondly, which other factors are associated with these preferences? Across two experiments, we measured how participants preferred to deploy both *attentional breadth* (using an adapted Navon task) and *eye movements* (using a free-viewing task). We also measured participants’ working memory capacities (Experiment 1), and their personalities and world beliefs (Experiment 2). In both experiments, there were consistent individual differences in preference for attentional breadth and eye movement characteristics, but these two kinds of preference were unrelated to each other. Working memory capacity was not linked to these preferences. Conversely, the personality trait of Openness to Experience robustly predicted two aspects of eye movement behavior preference, such that higher levels of Openness predicted *smaller* saccades and *shorter* scan paths. This suggests that personality dimensions may predict preferences for more absorbed engagement with visual information. However, it appears that individuals’ preferences for shifts of attention during scene viewing do not necessarily relate to the breadth of attention they choose to adopt.

**Supplementary information:**

The online version contains supplementary material available at 10.3758/s13414-024-02937-z.

## Introduction

Given the disparity between the vast amount of visual information offered by the world around us, and our limited perceptual and cognitive capacities, visual attention is a crucial mechanism for determining which parts of this information are selected for further processing. A core component of this selective mechanism is *spatial attention*, or the allocation of attention to specific subregions of the visual field. We deploy spatial attention to different locations in two ways: firstly, we can alter the spatial extent of the attended region (also called *attentional breadth*) around the point of attentional fixation (Feldmann-Wustefeld & Awh, 2019; Muller et al., 2003; Sasaki et al., 2001), and secondly, we can shift the spatial location of this fixation around the visual field (Kustov & Robinson, 1996; Sauseng et al., 2005; Yantis et al., 2002). For example, one might shift a narrow breadth of attention between the faces in a crowd to search for a friend, while a broader breadth of attention could be used to estimate the size of the crowd.

### Distinguishing preference and ability in deployments of spatial attention

As seen in this example, both kinds of spatial deployment can be made in response to contextual factors such as task-specific objectives. However, another focus in more recent literature has been the different *preferences* that individuals show for how they deploy spatial attention. These preferences become especially salient in circumstances where task requirements do not clearly compel a specific attentional deployment as the most efficient strategy (Caparos et al., 2013; Koldewyn et al., 2013; Wyche et al., 2023). Although some boundary issues arise from the imposition of such a dichotomy, we broadly define ability measures as those where task performance requires or benefits from the adoption of a specific strategy for deployment of spatial attention. Conversely, for a measure of preference, task demands are less salient or even absent altogether, and therefore performance reflects the preference or tendency to deploy spatial attention in a certain way. For instance, examples of tasks that gauge the ability to regulate spatial attention include those that require the participant to maintain a specific attentional breadth, and those for which the most efficient pattern of performance can only be obtained through a specific pattern of shifts (e.g., attentional orienting paradigms, or serial shifts of attention around a memory array to encode items). Conversely, examples of tasks that gauge preferences include those where either a broad or a narrow breadth of attention could equally be used, and those where there is no clear optimal strategy of attentional shifts (e.g., inspection of a complex naturalistic scene for delayed recall).

The dissociation of preference and ability in the deployment of attentional breadth is well-documented, as these two aspects have been explicitly compared in different contexts. For example, Caparos et al. (2013) were able to demonstrate this dissociation in a cross-cultural context comparing British and Himba observers. Although Himba participants demonstrated a preference for a narrower attentional breadth when the task did not require or benefit from adopting a specific breadth of attention, they were also more efficient at instantiating both narrow and broad breadths of attention in tasks where these respective breadths were required for optimal performance. Similarly, Koldewyn et al. (2013) found that while autistic children demonstrated a subjective preference for a narrow attentional breadth relative to their neurotypical peers, these two groups did not differ in their objective performance for a task that required the use of a broad attentional breadth. Preference and ability are therefore clearly dissociable for deployments of attentional breadth: tendency to adopt a certain breadth of attention when given the freedom to do so does not appear to constrain ability to adopt specific breadths of attention which are optimal for attainment of task objectives.

The dissociation of preference and ability in the deployment of attentional shifting behavior is not as well-documented as for attentional breadth, but there is some evidence consistent with it. Eye tracking is the most useful way to measure attentional shifting preferences, as eye movements cannot occur without a corresponding re-orientation of the attentional focus (Shepherd et al., 1986). Key preference metrics derived from eye tracking include fixation duration (how long fixations are held during image viewing), saccade amplitude (the length of saccades made during image viewing), scan path length (the summed length of all saccades made when viewing an image), and exploratory breadth (the spatial extent of the region over which saccades are being made). For instance, Van den Driessche et al. (2019) administered a visual search task to children who had also been given an ADHD assessment. While children with higher ADHD scores demonstrated a *preference* for saccades that were on average longer but also more variable in length, there was no difference in target location *ability* associated with ADHD score.

While the evidence tends to show that preference and ability are dissociable for behavioral measures of both attentional breadth and attentional shifts, a major unanswered question is whether preferences are related across these two different *kinds* of spatial-attentional deployment. In other words, is there a relationship between the attentional breadth that individuals prefer to use (i.e., attentional breadth preference), and the characteristics of the eye movements that they tend to undertake (i.e., attentional shifting preference)? Such a question is of major theoretical and applied interest because it sheds light on whether we have fundamental tendencies in how we acquire information from the visual world. This has implications not only for understanding individual differences in human cognition, but also for applied contexts such as assessing and training driver safety (Ahlström et al., 2021; Konstantopoulos et al., 2010; Wolfe et al., 2020). Associations between the ability to regulate both attentional breadth and eye movement behaviors have been found within specific task designs (Gaspar et al., 2016; Nuthmann, 2013; Wu & Wolfe, 2022), but evidence about how these deployments of spatial attention relate more broadly is sparse. Weber et al. (2000) found that when participants were instructed to process local features of a stimulus, saccades were executed in approximately half of trials, showing that this task could be executed either via covert or overt shifts of attention. Conversely, when instructed to process global features, participants made almost no saccades, indicating reliance on a broader breadth of attention to integrate the overall form of the figure being viewed. This finding suggests a relationship between attentional breadth and eye movement strategies, such that one of these two methods of information acquisition is prioritized in response to task demands.

However, while this study shows an interrelationship between attentional breadth and attentional shifts in an *ability*-based context, this may reflect optimization of behavior to conform to task demands. Extrapolating from Caparos et al.’s (2013) and Koldewyn et al.’s (2013) findings, it is possible that the ability to engage in this trade-off strategy may be dissociable from overarching *preferences* for how these two different types of spatial-attentional deployments are used in combination. A core purpose of this study is therefore to assess whether there are consistencies in how individuals choose to deploy their attention for modulations of both the point of spatial fixation, and the size of the attended region around the fixated point. It will also consider potential predictors of these preferences, the rationale for which is explored below.

### Factors associated with spatial-attentional preferences

When investigating the possibility of shared patterns of preference across attentional breadth and shifts, it is important to acknowledge that other factors may underlie or show associations with these patterns. Across two experiments, we considered some factors which may relate to any shared variance found: in Experiment 1, working memory capacity (WMC), and in Experiment 2, personality and environment beliefs. WMC has been shown to relate to the *ability* to regulate both attentional breadth (Bleckley et al., 2003; Goodhew, 2020b; Heitz & Engle, 2007; Kreitz et al., 2015) and eye movement behavior (Luke et al., 2018; although see Loh et al., 2022 for a discussion on how different aspects of working memory functioning are implicated in eye movement behavior), and there is some evidence it may be related to preference (Hayes & Henderson, 2017; Luke et al., 2018). Specifically, it is known that higher WMC is linked to longer fixations in a scene-viewing task, which the authors tentatively attribute to high-WMC individuals using a larger breadth of attention (Luke et al., 2018). In Experiment 1, therefore, we also included a measure of working memory capacity to investigate whether this may account for any shared variance in preference strategies across attentional breadth and shifts.

More broadly, if individuals show systematic preferences for how they deploy spatial attention, might these preferences relate to other characteristics which powerfully shape our experiences of daily living, such as our personalities and beliefs about the world? Some evidence suggests that personality may not only influence our evaluations of the world, but also how we tend to acquire information from the visual environment. For instance, Wilson et al. (2016) found that higher levels of trait Openness to Experience (Openness) were associated with the use of a broader attentional breadth. Swift et al. (2020) subsequently observed that higher levels of trait Conscientiousness predicted individual tendencies to show a global-precedence effect in a hierarchical-figures task requiring subjective judgments, a pattern of performance which has been interpreted to reflect a broader breadth of attention (Goodhew, 2020a). Regarding eye movement behavior, one study has found that higher levels of Neuroticism and Openness were both predictors of longer fixation durations in a short task where participants viewed two abstract animated videos (Rauthmann et al., 2012). Contrary to this, Hoppe et al. (2018) found that eye movement behaviors can be used to predict every Big Five personality trait *except* Openness. Altogether, while there is clear preliminary evidence that links personality with deployments of spatial attention, there are mixed findings regarding how specific traits relate to different kinds of deployment. Here, we sought to clarify this question in a large, well-powered study with measures optimized to selectively operationalize preferences (rather than ability) for attentional breadth and shifts.

Finally, recent work suggests that individuals’ fundamental beliefs about their environment (e.g., about whether the world is safe versus unsafe, or good versus bad), can have a formative influence on personality traits and can even outperform personality traits in predicting important outcomes such as well-being (Clifton et al., 2019). A recent measure of environment beliefs is the Primal World Beliefs scale (PI-99; Clifton et al., 2019), an extensively validated questionnaire which gauges an individual’s overarching beliefs about whether the world is *good*, as well as secondary beliefs about whether the world is *safe*, *enticing*, and *alive*. While it is easy to make semantic connections between these world beliefs and possible spatial-attentional preferences (e.g., that individuals who believe that the world is a safe and interesting place will demonstrate a preference for more exploratory attentional behaviors), there is currently no evidence about the relationship between these core environment beliefs and spatial-attentional functioning. We therefore included the PI-99 in Experiment 2 as an exploratory test of whether environment beliefs relate to deployments of spatial attention.

### Present study

This study was exploratory in nature: therefore, instead of specifying hypotheses about the relationships between variables of interest, we formulated two aims. The primary aim of the present study was to investigate whether preferences for spatial-attentional deployments are related across different *types* of deployment, namely attentional breadth and shifts of attention. Across two experiments we used a variant of the Navon task adapted to measure individuals’ preferences regarding the size of their attentional breadth, and a nature scene viewing task where eye movements were measured to gauge preferences in the spatial extent of eye movement. In addition, we aimed to assess whether other factors of interest were linked to preferences for the deployment of spatial attention: in Experiment 1 we measured individuals’ working memory capacity, while in Experiment 2 we measured individuals’ personality and their fundamental beliefs about the world.

## Experiment 1

In Experiment 1, a large sample (*N* = 135) of participants completed two measures of attentional breadth preference (a Kimchi-Palmer task and an undirected Navon task), as well as a measure of eye movement behavior preference (a free-viewing task with delayed recall). Participants also completed a measure of WMC (the Automated Operation Span Task; AOSPAN). After checking the validity and reliability of key measures of interest, the relationships between (a) attentional breadth preferences and eye movement behavior preferences, and (b) all spatial-attentional measures and WMC, were assessed via a correlational analysis.

### Methods

#### Participants

Sample size was determined based on a priori power analysis conducted using the bivariate correlation function in G*Power (Faul et al., 2007). The selected method of analysis was two-tailed Pearson correlation, such that there was 90% power to detect a Pearson correlation of 0.3, a modest effect size according to Cohen’s (2013) conventions. Consequently, the appropriate sample size was determined to be 112 participants; an extra 20% allowance for exclusions and technical issues was factored into this sample size, so 134 participants were sought. Ultimately, 135 participants completed the study as it was slightly overenrolled to ensure that data collection deadlines were met. Eligibility criteria for participation were an age of between 18 and 40 years inclusive, and normal or corrected vision.

For these 135 participants, mean age was 20.88 years (SD = 3.11 years). Thirty-nine participants were male, 93 participants were female, and three participants were non-binary. One hundred and nineteen participants reported being right-handed, and 16 left-handed. Twenty-seven participants reported wearing contact lenses, 24 participants reported wearing glasses, while the remaining 84 participants had normal vision. For country of birth, 56 participants reported being born in Australia, 55 participants reported an East Asian country (e.g., China, South Korea, Taiwan), ten reported a South Asian country (e.g., Bangladesh, India, Pakistan), four reported a Southeast Asian country (e.g., Malaysia, Singapore, Vietnam), while ten reported being born in another country (e.g., UK, USA, South Africa). All participants provided written informed consent before participation and were able to withdraw from participation at any time without penalty. The Australian National University’s Delegated Science and Medical Human Research Ethics Committee approved all ethical aspects of the experiment (Protocol 2022/119).

#### Materials and procedure

Participants were tested individually in a laboratory setting. All stimuli were displayed on a 24-inch BenQ XL6430T monitor with a refresh rate of 60 Hz, with all stimulus presentations and response recording done using MATLAB’s Psychophysics toolbox (Brainard, 1997), except AOSPAN in Experiment 1, which was administered using Inquisit 6.6.1. Eye movement data were recorded using a desktop-mounted Eyelink 1000 Plus eye tracker, which sampled the left eye at 1000 Hz. Participants viewed the screen from a distance of 80 cm, using a fixed chinrest to ensure that their heads remained stable during eye tracking.

After informed consent was obtained, a survey was administered to collect participants’ demographic information, as well as their responses to the Kimchi-Palmer task, a subjective measure of attentional breadth preference. Participants then completed three tasks using a fixed running order. This fixed running order was chosen for two reasons: firstly, to avoid introducing between-participant variance stemming from different running orders, given that between-participant differences were the focus of analysis, and secondly because many participants find the final experimental task (AOSPAN) to be lengthy and mentally taxing, and we did not want any negative emotion which arose during its completion to affect participants’ performances on other tasks (Laybourn et al., 2022). Firstly, tasks designed to measure preferences for (1) attentional breadth (Navon task) and (2) eye movement behavior (free viewing task with delayed recall) were administered. Participants were given task instructions by the experimenter, then completed a practice block (a small number of sample trials with feedback provided). If a performance threshold of 75% accuracy was not met, participants repeated the practice task until able to pass it, and then progressed to completion of the experimental block. Finally, participants completed (3) the Automated Operation Span Task (AOSPAN), a standardized measure of working memory capacity. After completing the three tasks, participants were then debriefed about the purpose of the experiment. Experiment sessions took approximately 60 min on average, and participants were compensated with either a cash payment ($20AUD) or research participation credit for relevant courses.

#### Experimental tasks

##### **Attentional Breadth: Kimchi-Palmer Task**

**Fig. 1:**
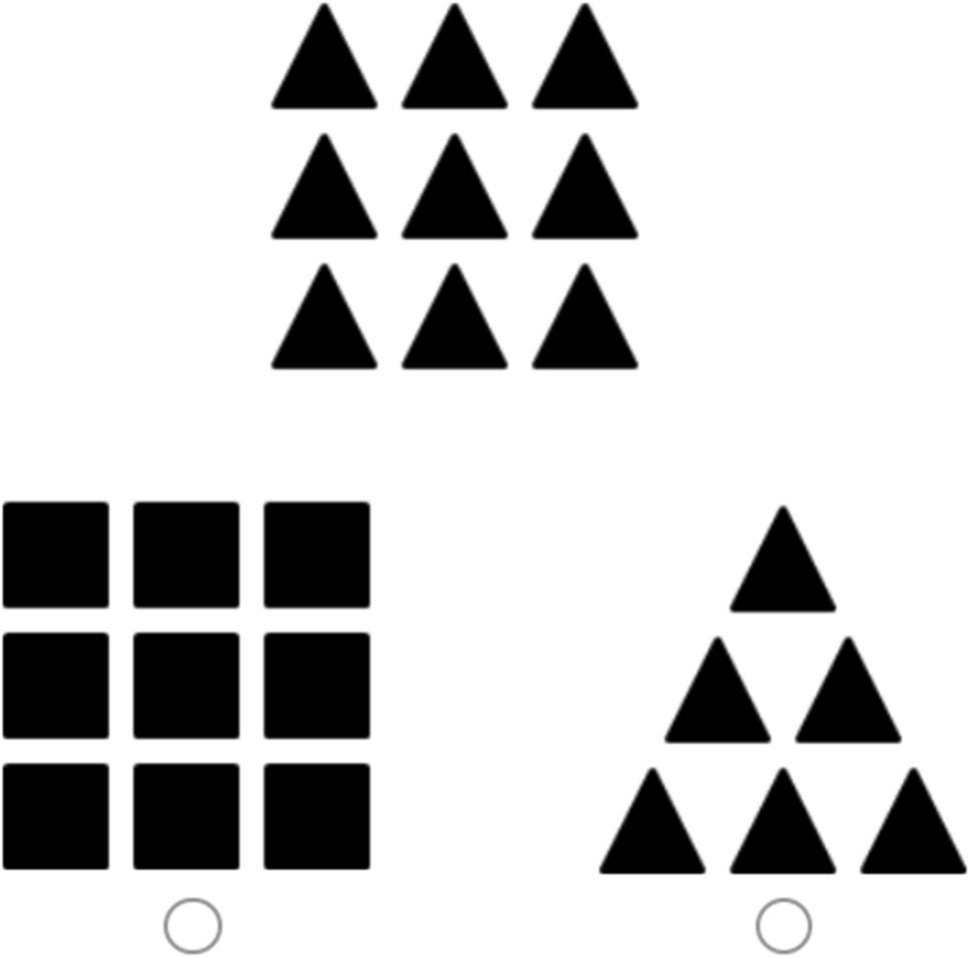
Sample Trial for Kimchi-Palmer Task

The Kimchi-Palmer task (Kimchi & Palmer, 1982) uses hierarchical configurations of shapes to gauge participants’ subjective preference for a relatively broad or narrow attentional breadth. In this task, participants are presented with a reference shape configuration (e.g., three small triangles arranged in the shape of a larger triangle), as well as two other comparison configurations which are presented below the reference configuration. The participant is instructed to choose which of these two comparison configurations they consider to be the most similar to the reference configuration (see Fig. 1). Of the two comparison options, one is consistent with the reference configuration at the large or global level (e.g., a large triangle made up of small squares), while the other is consistent with the reference configuration at the small or local level (e.g., a large square made up of small triangles). The participant’s choice from these two available options is understood to indicate whether the global or the local level of the test stimulus was more salient to them and, therefore, whether they had a broad or narrow attentional breadth.

In the present study, participants completed six trials, with a binary scoring system for each trial: if the reference configuration was rated as being more similar to the local-consistent comparison stimulus, the trial was scored as 0, while if it was rated as being more similar to the global-consistent comparison stimulus, the trial was scored as 1. Final scores for the task thus ranged from 0 to 6, with a higher score indicating a greater preference for a broad breadth of attention.

##### **Attentional Breadth: Navon Task**

To complement the subjective self-report measure of attentional breadth preference obtained using the Kimchi-Palmer task, we used mean response time to identify the target in an undirected version (Goodhew, 2020a) of the Navon hierarchical-letters (Navon, 1977) task as a more objective measure of attentional breadth preference. In a Navon task, participants are presented with a hierarchical letter (a repeated small capital letter arranged to form the shape of a large capital letter) on each trial. Neural-imaging evidence shows that participants adopt different breadths of attention when identifying the different components of Navon stimuli: when attending to the *local* level (the small, repeated letter), they adopt a relatively small attentional breadth, but when attending to the *global* level (the large, overall letter shape), they adopt a relatively large attentional breadth (Sasaki et al., 2001).

In the traditional (or *directed*) version of the Navon task, one of two target letters always appears at the level of detail which participants are instructed to view: either local or global. At the level of detail to which the participant is not actively attending, the letter(s) can either be congruent or incongruent with the target, and the level of interference from this non-attended level is used to gauge the participant’s success in adopting the attentional breadth required for the task. Linking back to the dichotomy of preference versus ability established in the Introduction, the directed Navon task can therefore be considered an *ability* measure (Goodhew, 2020a): it operationalizes participants’ ability to adopt a specific strategy of regulating or controlling attentional breadth to focus exclusively on the relevant level of detail when attempting to identify the target letter in the figures being presented.

Conversely, the undirected version of the Navon task can be considered a *preference* measure, because it reflects the attentional breadth that individuals tend to use when there is no performance advantage conferred by continuously adopting one specific attentional breadth. In an undirected Navon task, participants are not told to attend to a specific level of the figures shown to them. Instead, their task is to identify which of multiple possible target letters is present in the stimulus, and the target letter has an equal chance of appearing at either the global or the local level of detail on each trial. Further, unlike the directed Navon, a target only appears at one level of a stimulus, with a non-target letter appearing at the other level of detail. The key measure of interest for an undirected Navon task is a difference score comparing mean response times for trials where the target appeared at the local level to trials where the target appeared at the global level. This measure gauges attentional breadth preference (Goodhew, 2020a): if an individual tends to adopt a narrow attentional breadth when all other factors are held equal, then they must resize to a larger breadth of attention on target-global trials, incurring an RT cost. Therefore, an RT advantage for these local trials would indicate a greater tendency to adopt a narrow breadth of attention.

In our undirected Navon task, participants were asked to identify which of two target letters (H or T) was present in the stimulus shown on each trial as quickly and as accurately as possible by pressing the H or T key on a standard keyboard. Eight stimuli were used (four target-local, four target-global), such that each had an equal probability of appearing on each trial and the target letter was present at only one level of detail. Using the nomenclature of GLOBALLETTER-LocalLetter, the eight stimuli were: Te, Tf, He, Hf, Et, Ft, Eh, and Fh. Global figures subtended 3.5 by 3.5 degrees of visual angle, while each local letter subtended 0.6 by 0.6 degrees. Navon figures were black, the letters were always uppercase, and figures were presented centrally on a white background.

**Fig. 2:**
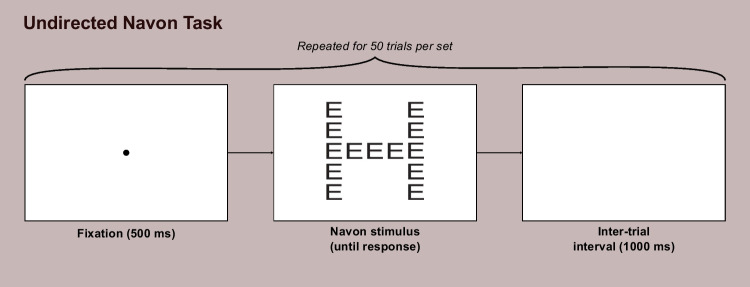
Trial Procedure for Undirected Navon Task. *Note.* Participants completed four sets of trials overall

The trial procedure for our undirected Navon task is shown in Fig. 2. After a fixation dot was presented in the center of the screen for 500 ms, a Navon stimulus appeared and remained visible until a keyboard response was entered. RTs were recorded as the time elapsed from the onset of the Navon figure’s display to the keyboard response. After a response was entered there was a 1000 ms inter-trial interval in the form of a blank screen, after which the next trial began. Participants completed four sets of 50 trials each, with a rest break terminated at the participant’s discretion offered after finishing each set.

##### **Eye movement behavior: Free viewing task with delayed recall**

**Fig. 3:**
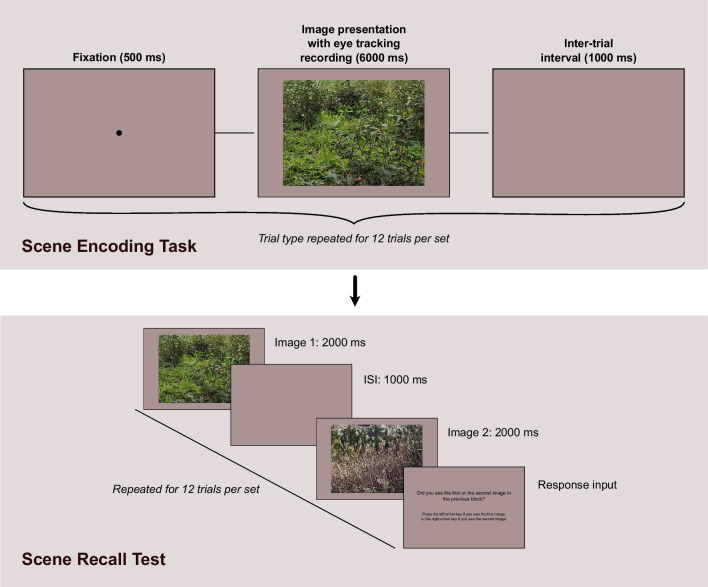
Trial Procedure for Scene Recall Task. *Note.* Participants completed four sets of trials overall

To assess individual differences in preference for shifts of attention, we used a free viewing task which required delayed recall of the scenes being viewed (Cronin et al., 2020a, b; Loh et al., 2022; Wyche et al., 2023), such that we tracked participants’ eye movements as they viewed nature scenes with the knowledge that they would have to later recall them during a memory test. This task design is a good measure of preference or tendency behavior for eye movements: unlike other tasks which require fixation of specific objects such as foraging or cueing paradigms, memorizing complex naturalistic scenes for later recall leaves many information acquisition strategies open to participants. Indeed, evidence from the use of these paradigms shows that different individuals adopt different patterns of eye movements around these visual scenes, and that scene-based variance in eye movement behavior is minimal (Wyche et al., 2023). The inclusion of a delayed memory test for recall of the viewed scenes serves two purposes. Firstly, it reduces the likelihood of demand characteristics affecting eye movement behavior when participants know that their eye movements are being tracked. Secondly, it provides a measure of recall ability, allowing evaluation of whether this is associated with preference for specific eye movement behaviors Fig. 3.

**Fig. 4:**
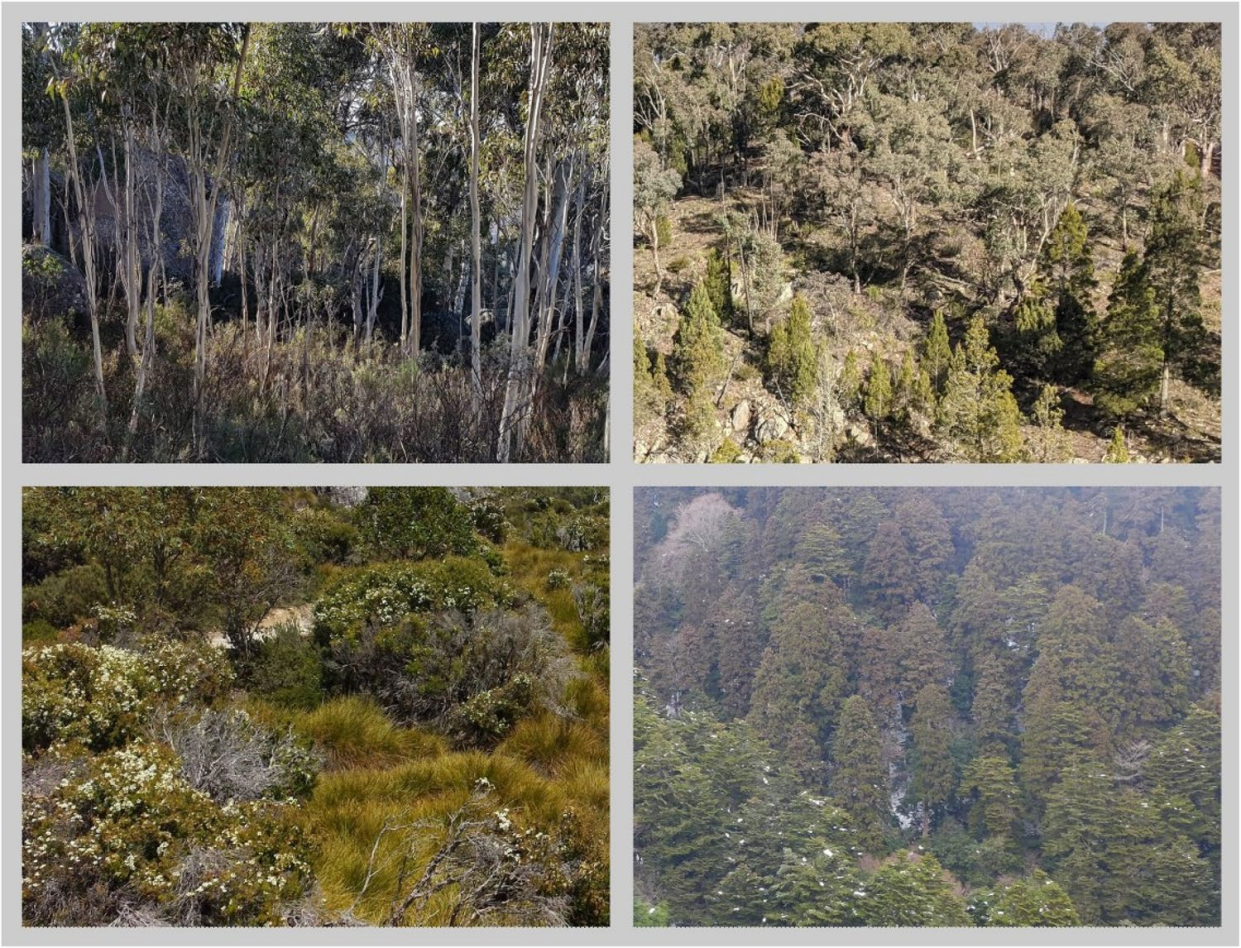
Examples of Stimuli Used in Scene Recall Task

The free viewing task used a set of 96 photographs of natural scenes, which were characterized by even distributions of equally salient objects across the scenes. This design choice avoided the presence of any dominant image elements in the scene composition, which could (a) skew the gaze metrics of interest (discussed below) by attracting and holding viewers’ attention, and (b) provide a heuristic for task completion, namely quickly memorizing salient objects from each array and terminating active visual inspection, rather than attempting to memorize the scene in its entirety. Examples of these images can be seen in Fig. 4. Participants completed four sets of 12 scene-viewing and memory recall trials each with a rest break after each set, for a total of 48 trials. All participants saw the same set of 48 images in a randomized order during the eye tracking portion of the task, with the other 48 images being randomly used as alternate response options in the memory recall trials (see below).

The trial procedure is illustrated in Fig. 3. Prior to each scene-viewing set, participants underwent Eyelink’s standard 9-point eye-tracking calibration procedure. Each scene-viewing trial began with the presentation of a fixation dot in the center of the screen for 500 ms. After this, an image presented in landscape orientation subtending 22.5 by 17.0 degrees of visual angle was presented for 6000 ms against a grey background (RGB: [147, 147, 147]). At the end of the image presentation, eye movement data recording was terminated, and a blank screen was shown during a 1000 ms inter-trial interval.

For each set, after completion of the 12 scene viewing trials, participants then completed 12 memory recall trials. On each memory recall trial, two images were shown consecutively for 2000 ms each, with a 1000 ms blank screen between: one was an image which had been seen during the prior scene-viewing block, while the other was a different image which had not been seen before. Participants were asked to identify whether they had seen the first or second image in the previous scene-viewing block, pressing the left arrow key on a standard keyboard if they had seen the first image, and the right arrow key if they had seen the second image. Whether the first or second image was the one that they had seen before was randomized, and there was no systematic correspondence between the trial number on which an image was shown in the scene-viewing block, and the trial number on which it appeared in the memory recall block. There was no repeat of images across any of the trial blocks.

We extracted four metrics of interest for each individual participant to investigate the spatiotemporal dynamics of exploratory behavior:

- Event-level means and standard deviations of fixation duration (i.e., all fixation durations were analyzed for each participant without averaging at trial level)
- Event-level means and standard deviations of saccadic amplitude
- Event-level exploratory breadth (Euclidean distance of each fixation from image center)
- Trial-level scan path length (sum of all saccadic amplitudes on each trial)

Scan path length was included as a trial-level variable to provide a different level of granularity from the other event-level variables (Castelhano et al., 2009; Cronin et al., 2020a, b; Wyche et al., 2023). Event-level analysis can provide powerful insights into individual differences in eye movement behavior, but it does not reflect how viewing periods are segmented into trials, and thus information about how fixation duration and saccadic amplitude interact over the trial period can be lost. By representing information about how eye movements were made over the key time period of interest (the trial), the metric of scan path length concisely reflects how much exploration of the visual scene each participant tended to undertake during the time available to them. Therefore, event-level variables and trial-level scan path lengths provide qualitatively different insights into individual differences in eye movement behavior.

##### **Working Memory Capacity**

**Fig. 5:**
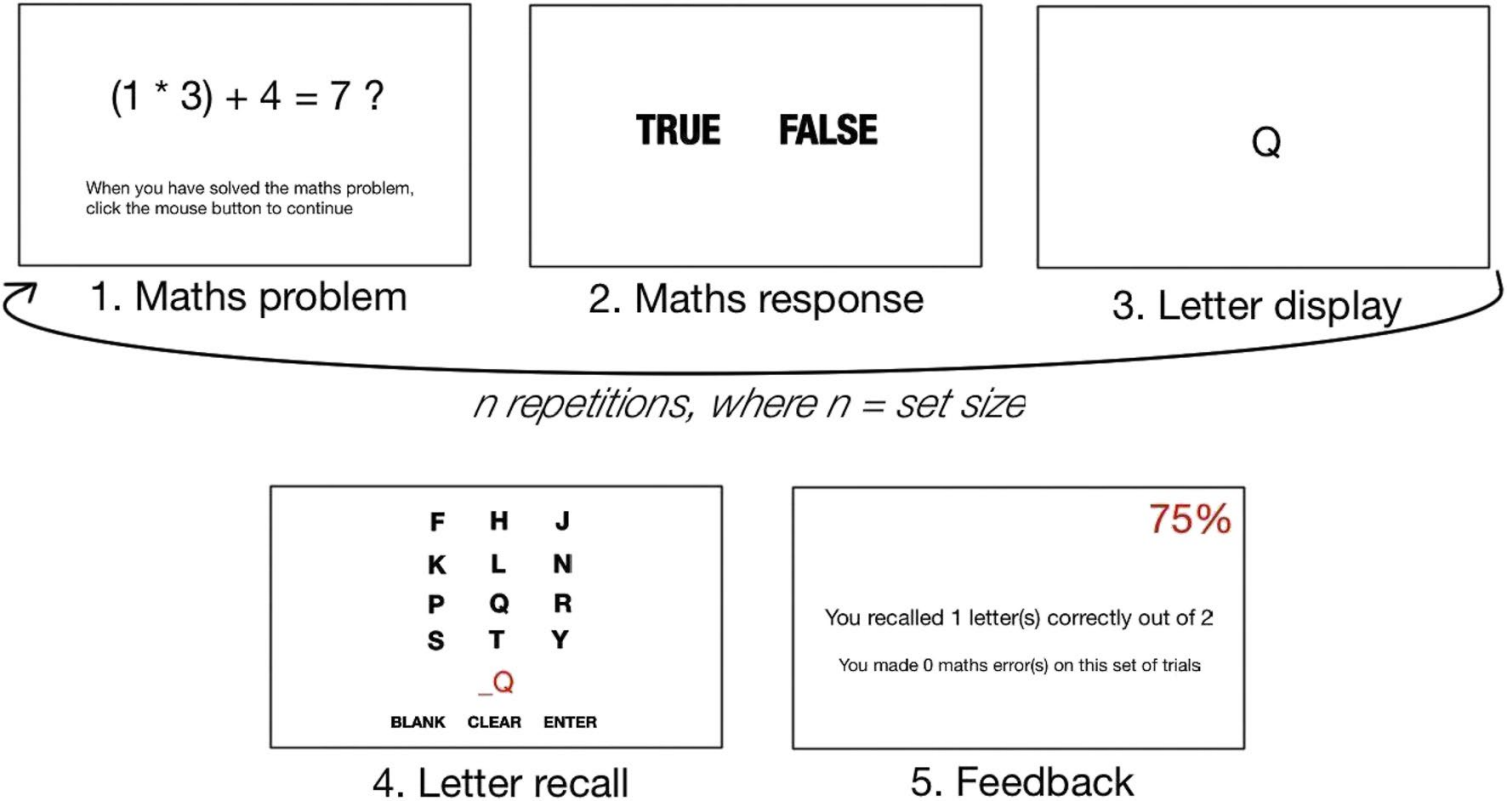
AOSPAN Task Procedure. *Note.* Letter recall and feedback screens were shown after all equation-letter combinations in a set were shown. The red number on Screen 5 (Feedback) is the cumulative accuracy score for math trials

To measure working memory capacity (WMC), we used AOSPAN (Unsworth et al., 2005), a standardized and well-validated measure. AOSPAN requires participants to solve a sequence of simple math problems, which are interleaved with a set of letters that must be recalled at the end of the sequence. For each trial, the participant must solve a mathematical problem and then recall a letter shown immediately after. These equation-letter combinations are presented between three and seven times; the number of presentations in a sequence is called the *set size*. Three presentations of each set size are used, for a total of 75 equation-letter combinations, and the ordering of the set sizes is randomized to prevent the number of items in a set from becoming predictable. Following each set, the participant is instructed to recall the letters in the order presented, after which they receive feedback about the number of letters which were correctly recalled and the number of errors they made for the math problems. To prevent participants from focusing exclusively on letter recall, an 85% accuracy criterion on the math problems is imposed, and participants also see their cumulative accuracy percentage on each feedback screen (Unsworth et al., 2005). The trial procedure for AOSPAN is shown in Fig. 5.

The task begins with a practice of the letter recall component followed by a practice of the math component, to introduce participants to the mouse-based response system. During the math practice, the RT for each problem is recorded and an average is calculated for each participant to account for individual differences in the time taken to solve math problems (Unsworth et al., 2005). This average RT plus 2.5 SDs is used as a time limit for the math displays in the experimental session. In the final practice session, the participants perform the math problem and letter recall components together as equation-letter combinations. For the math problems, if participants do not respond within the time limit, the task records a *speed error* and progresses to displaying the letter. This prevents participants from rehearsing the letters instead of solving the math problems (Unsworth et al., 2005). After completing the practice sessions, participants complete the experimental session. A letter response was recorded as correct if a participant entered a correct letter item in the correct sequence position on the recall screen. Throughout the experiment session, participants were monitored for task compliance by the experimenter to ensure they were not using alternate strategies for letter recall such as repeating out loud the sequences of letters that had been viewed.

Five outcome measures were calculated: (1) Operation Span (OSPAN) score, (2) total letters correct, (3) math errors, (4) speed errors, and (5) accuracy errors. OSPAN score is the sum of all perfectly recalled letter sets, while total letters correct is the sum of all correctly recalled letters. For instance, if 3 letters were correctly recalled in a set size of 3, 4 in a set size of 4, and 3 in a set size of 5, the OSPAN score would be 7 (3 + 4 + 0), while the total letters correct score would be 10. ‘Math errors’ represents the total number of errors in solving the math problems, combining speed errors and accuracy errors (when the participant solved the problem incorrectly).

The key operationalizations for working memory in AOSPAN are OSPAN score and total letters correct, although total letters correct is recognized as the more sensitive measure (Conway et al., 2005; Redick et al., 2012). Math error scores serve as a data quality screening tool, ensuring that participants have not used a strategy of neglecting math problems to achieve high letter recall scores. Higher letter recall scores indicate higher working memory capacity, as they indicate that more information can be held in working memory while functioning under high cognitive load (Engle, 2002).

#### Data preparation

For the Navon task, RTs were subjected to trial-level screening for each participant: trials were removed from further analysis if participants responded too quickly (< 100 ms) or too slowly (> 2.5SDs above participant’s mean RT; Goodhew et al., 2020). This exclusion was designed to remove trials where RTs reflected either pre-emptive responses or task disengagement. Only a small number of trials were excluded for each participant on this basis (*M* = 2.9%, SD = 0.8%).

Eye movement data were extracted using Data Viewer for Eyelink (SR Research, 2017) and processed in MATLAB. Fixations and saccades were segmented in accordance with Eyelink’s standard algorithm using velocity (30°/s) and acceleration (9500°/s^2^) thresholds. Before further analysis, screening of eye movement data was also applied at trial and event levels to improve data quality. The entire datasets of participants with track loss rates of greater than 25% (*n* = 2) were excluded prior to event-level screening. For fixation data, fixations outside the bounds of the presented image, as well as those with extremely short (< 50 ms) or long (> 1500 ms) durations, were excluded, resulting in a loss of 4.1% of fixation datapoints. Saccades of greater than 28.2° (i.e., the diagonal dimension of the presented image), as well as those with zero and null values, were removed, resulting in a loss of 0.6% of saccade datapoints.

In eye tracking research, the fixation duration distributions of each individual participant typically show significant rightward skew, such that their distributions are characterized by a tail of longer fixation durations. A common response to this problem is the fitting of ex-Gaussian distributions to individual participants’ fixation duration distributions (Guy et al., 2020). An ex-Gaussian distribution is produced by combining a normal and an exponential distribution, such that the normal distribution is given a more heavily weighted rightward tail. Ex-Gaussian distributions can be described using three parameters: *mu* (the mean of the normal part of the distribution, indicating average performance for the majority of datapoints), *sigma* (the standard deviation of the normal part of the distribution, indicating the spread of scores for the majority of datapoints), and *tau* (the exponential decay function, indicating the weighting and spread of the rightward tail of outlier fixations). Using ex-Gaussian distributions to model fixation durations produces superior goodness-of-fit when compared to the use of Gaussian (normal) distributions (Guy et al., 2020). Therefore, following the fixation screening described above, ex-Gaussian distributions were fitted to each participant’s fixation duration data using the *timefit* function of the *retimes* package in R (Massidda, 2013).

### Results

#### Overview

Raw data for this study are available at this link: https://osf.io/aqdb4/. This Results section is divided into the following subsections: participant exclusions are summarized, experimental effects and task reliability are considered, and finally correlations between variables of interest are assessed.

#### Participant exclusions

Sample size was calculated at *n* = 134 (see Methods section), and 135 participants ultimately took part in the study. After 18 participant exclusions following screening for outliers, the final sample size was *n* = 117, which exceeded the required power for intended statistical analyses. Any participant who was excluded in a specific step of data screening was not counted again when they met an exclusion criterion in subsequent steps.

For the Navon task, the main dependent variable of interest was RT on correct-response trials, calculated after RT-based screening of each participant’s data (see Methods section). No participant had more than 10% of trials excluded by the RT-based screening procedure, so no exclusions were performed on this basis. A minimum criterion of 80% accuracy in all conditions was applied to ensure compliance with task instructions; no participants were excluded on this basis. Finally, univariate outlier screening was performed using a z-score criterion (± 3.29, *p* < 0.001) for RT (Tabachnick & Fidell, 2013). Four participants’ data were excluded on this basis.

For the free viewing task with delayed recall, data screening and fitting of ex-Gaussian distributions for fixation duration data were undertaken as described in the Methods section. As described in the ‘Data Preparation’ subsection above, two participants had track loss rates of greater than 25% for their eye movement recordings, so their data were excluded on this basis. One participant performed below 66.67% accuracy for the memory probe task, so this participant’s data were excluded on this basis. The same z-score criterion used for Navon outlier screening was applied to individual participants’ screened means for fixation duration, saccade amplitude, exploratory breadth (Euclidean distance of fixations from center), and scan path length; one participant’s data were excluded on this basis. The fitting of an ex-Gaussian distribution to fixation duration data failed to converge for two participants, whose data were excluded from all analyses on this basis.

When administering AOSPAN, it is convention to exclude any participants who score under 80% accuracy in the math component of the task, as this may indicate that these participants preferentially attended to the letter recall component of the task and were thus less affected by the intending working memory load manipulation. The data of four participants were excluded on this basis.

Finally, given the individual-differences nature of this experiment design, any further participants who had missing data for one or more experiment condition were excluded. The data of a further four participants were excluded on this basis.

Overall, 18 participants’ data were excluded from further analysis, for a total sample of *n* = 117 in the final analysis. Application of less stringent screening criteria which did not exclude participants who had missing data meant that some additional correlations were statistically significant (see Supplementary Materials). However, the analysis reported below retained this exclusion criterion to ensure that the same sample was used for all analyses.

#### Distributions of experimental performance

Descriptive statistics for performance on our experimental tasks are reported in Table 1. Shapiro–Wilk tests (reported in Supplementary Materials) indicated violations of the assumption of normality for many key variables, so non-parametric tests were used for subsequent analyses. To ensure that robust individual differences were present, we also fitted linear mixed-effects models with random effects for participant and stimulus entered to all variables of interest (see Supplementary Materials). This analysis confirmed that not only did performance on all preference metrics vary meaningfully across participants, but also that this variance outweighed that which was driven by stimulus-specific features.

**Table 1:** Descriptive Statistics for Task Performance in Experiments 1 and 2

Variable	Exp 1		Exp 2	
	*M*	*SD*	*M*	*SD*
Kimchi-Palmer Score Navon Task	3.50	2.08	-	-
Global Accuracy (%)	98.10	2.12	98.86	1.40
Local Accuracy (%)	97.59	2.85	98.00	2.39
Global RT (ms)	631.76	139.45	643.55	175.62
Local RT (ms)	678.48	176.43	678.00	205.67
Preference Score (ms)	46.72	83.38	34.45	66.20
Free Viewing Task				
Memory Test (%)	94.80	5.30	95.20	5.00
Fixation Duration (mu)	160.46	23.08	155.87	24.66
Fixation Duration (sigma)	57.40	14.54	56.15	10.64
Fixation Duration (tau)	143.55	40.00	144.49	38.48
Saccadic Amplitude (°)	4.42	0.82	4.49	0.75
Exploratory Breadth (pixels)	248.98	36.09	255.84	40.88
Scan Path Length (°)	72.50	17.24	73.32	18.15
AOSPAN				
AOSPAN Score	48.04	16.24	-	-
Letters Correct	62.38	10.94	-	-
Math Total Errors	2.89	2.62	-	-
Math Speed Errors	0.87	1.18	-	-
Math Accuracy Errors	2.02	2.05	-	-
NEO-FFI-3				
Openness	-	-	43.55	5.87
Conscientiousness	-	-	42.39	7.80
Extroversion	-	-	40.80	6.00
Agreeableness	-	-	45.23	6.08
Neuroticism	-	-	37.59	9.33
PI-99				
Good (mean)	-	-	4.00	0.53
Safe (mean)	-	-	3.75	0.66
Enticing (mean)	-	-	4.37	0.64
Alive (mean)	-	-	3.53	0.76

Experiment 1 *n* = 117, Experiment 2 *n* = 121.

**Fig. 6:**
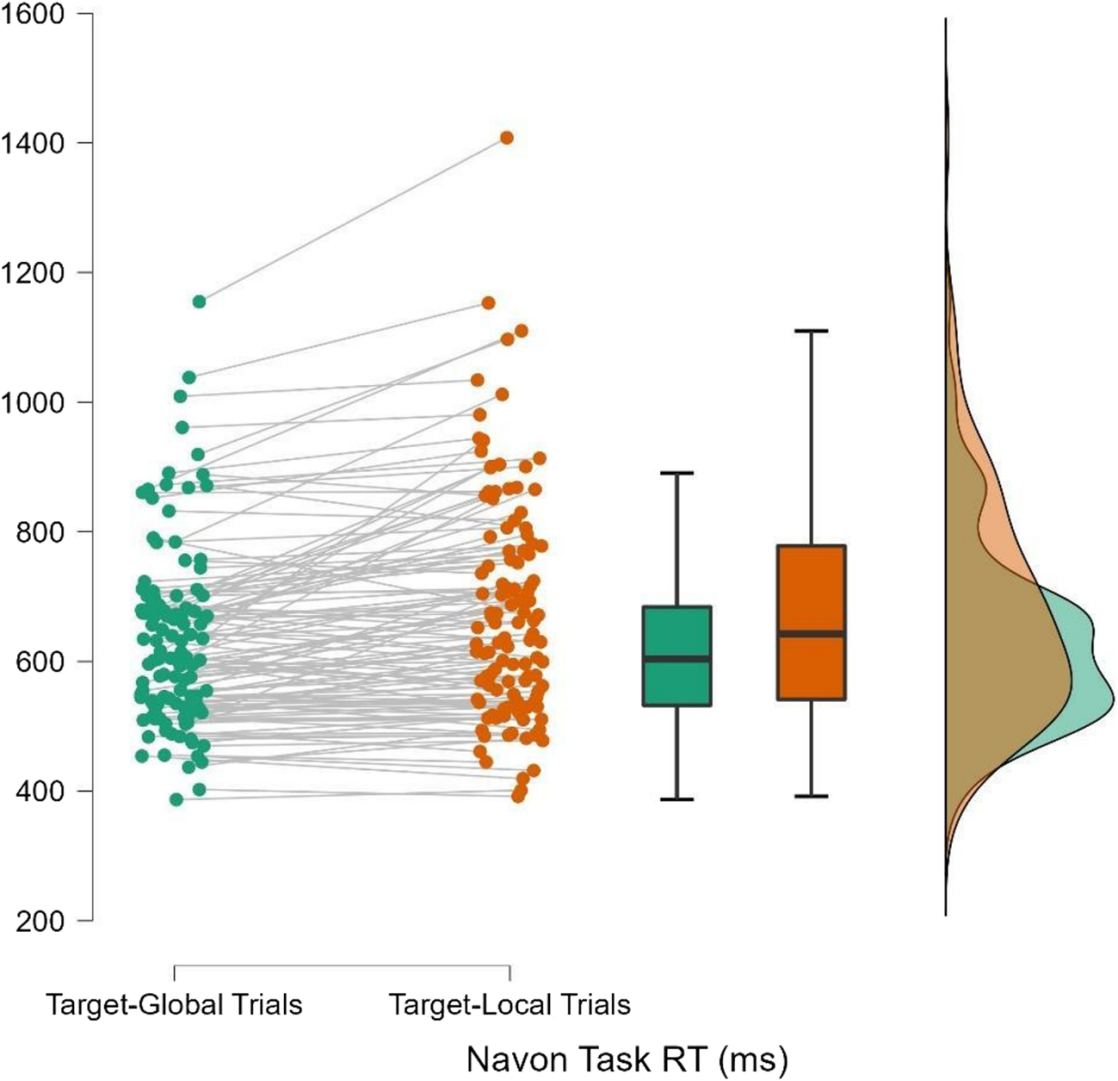
RT Performances by Participant for Navon Task in Experiment 1. *Note.* Performance slopes across Navon task conditions (target-global vs. target-local trials) are shown for each participant, as well as boxplots and distributions for sample performance in each condition. Performance in the target-global condition is shown in green, while performance in the target-local condition is shown in orange

RTs for the global and local trials of the Navon task were compared using a Wilcoxon signed-rank test (a non-parametric test was used as these RT scores were not normally distributed). Results indicated that participants’ RTs were faster for global trials than local trials; *z* = -5.37, *p* < 0.001, *r*_*rb*_^1^ = -0.572 (95% CI: [-0.696, -0.415]). This provides converging evidence for the external validity of our version of the Navon task. Even though there was significant individual variance in attentional breadth preference in the sample (see Fig. 6), the overall results are consistent with the global precedence effect traditionally observed with Navon stimuli, such that RTs are faster on target-global trials (Bar et al., 2006; Furtak et al., 2022; Navon, 1977).

Sample performance on AOSPAN was better than normative data for young adults (*n* = 6,326; Redick et al., 2012). OSPAN and Letters Correct scores in our experiment and the normative sample (*M*_OSPAN_ = 42.04; *M*_Letters Correct_ = 57.36) were compared using a one-sample Wilcoxon signed-rank test, using the mean score for the normative data as the test value. Results confirmed that our sample’s performance was better than that of the normative sample for both OSPAN (*z* = 3.807, *p* < 0.001, *r*_*rb*_ = 0.405, 95% CI: [0.218, 0.564]) and Letters Correct (*z* = 5.726, *p* < 0.001, *r*_*rb*_ = 0.610, 95% CI: [0.462, 0.725]) measures.

#### Task reliability

Task reliability (i.e., the stability of participants’ individual performance levels across time on the experiment tasks) was also examined.^2^ Reliable effects are crucial for further analysis in individual-differences designs, as reliability constrains the strength of any relationships that can be observed between tasks (Goodhew & Edwards, 2019; Hedge et al., 2018; Parsons et al., 2019; Spearman, 1910).

Reliability for the difference score measure calculated for the Navon task, as well as the eye movement behavior metrics from the free viewing task, were estimated with the R package *splithalf* (Parsons, 2020), which uses a permutation-based calculation of the correlation between scores derived from two halves of total trials (e.g., odd versus even trials) for all participants in the sample. This tool calculates the correlation between the two halves of trials over 5000 random splits of the trials. This approach provides a mean estimate of split-half reliability, as well as a 95% confidence interval around that estimate (see Table 2). This analysis indicated that all these metrics demonstrated excellent reliability.

**Table 2:** Spearman-Brown Corrected Reliability Estimates for Selected Tasks in Experiment 1

Task	*r*_SB_^a^	95% CI	
		Lower	Upper
Navon Preference Score	0.87	0.81	0.91
Free Viewing Task			
Fixation Duration	0.98	0.98	0.99
Saccadic Amplitude	0.98	0.97	0.98
Exploratory Breadth	0.98	0.97	0.98
Scan Path Length	0.96	0.95	0.97

^a^ Spearman-Brown corrected reliability estimate

*N* = 117

The reliability of AOSPAN can be calculated as an internal consistency measure. The number of letters recalled at the first, second and third presentations of each set size are combined into three sub-scores for which Cronbach’s alpha is computed (Unsworth et al., 2005). In our sample, the observed Cronbach’s alpha for AOSPAN was 0.833 (95% CI: [0.679, 0.956]).^3^ This indicates good reliability for AOSPAN as administered in this study (Tavakol & Dennick, 2011).

Overall, these assessments of task reliability indicated that our variables of interest were suitable for correlational analysis, as the observed strength of correlations between variables was unlikely to have been attenuated by poor measurement reliability.

#### Correlations

**Fig. 7:**
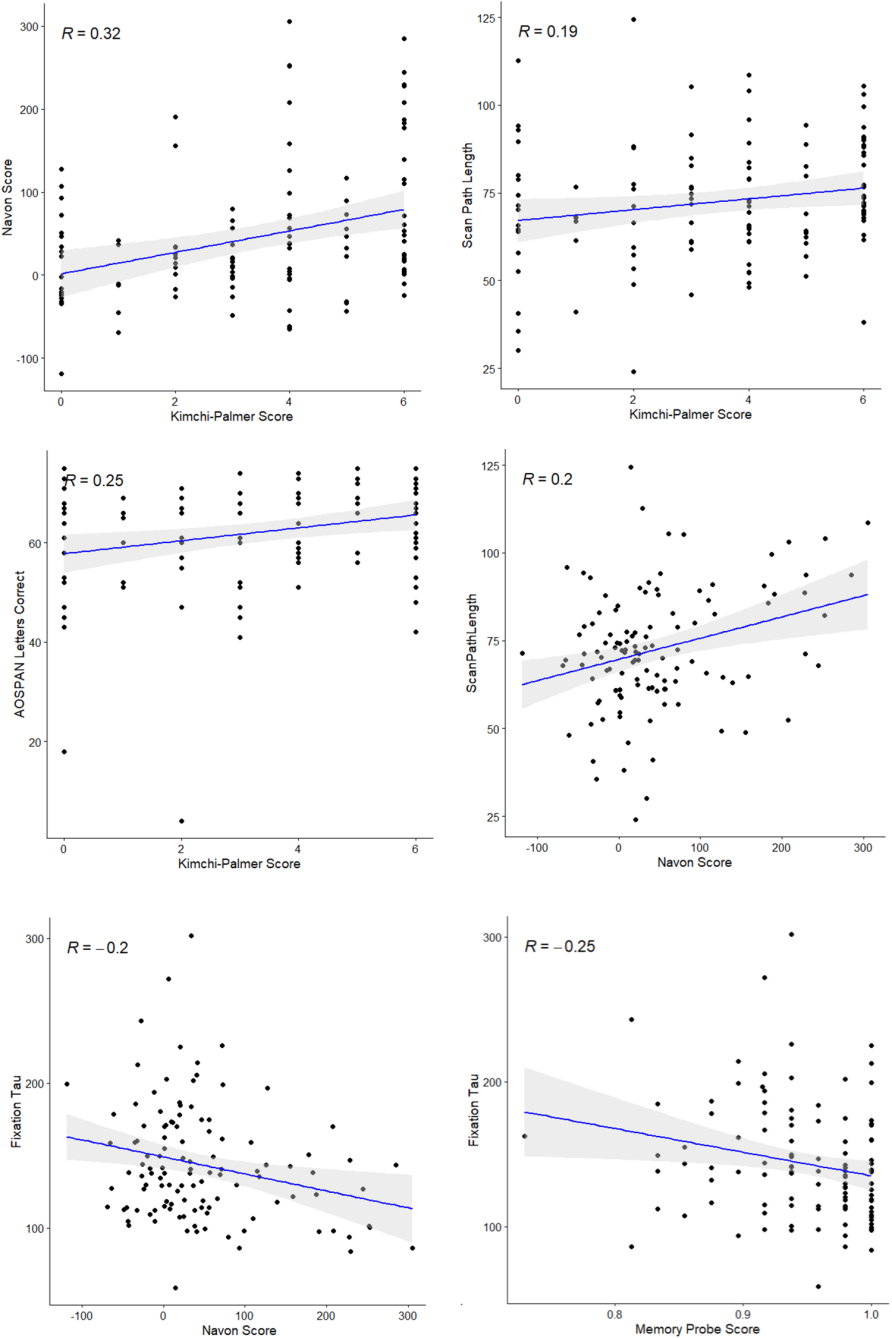
Scatterplots of Key Comparisons between Variables for Experiment 1. *Note*. Spearman correlations reported. Grey shaded area represents 95% confidence interval around regression line

Finally, we addressed the two research questions that motivated this study (whether preferences for spatial-attentional deployments are related across different types of deployment, and whether any shared variance in these preferences is linked to WMC) with a correlational analysis. The strength of evidence in support of these relationships was also assessed using the approach described in van Doorn et al. (2020), which permits Bayesian inference for Spearman rank correlation coefficients. Bayes factors are interpreted in accordance with the guidelines in Andraszewicz et al. (2014). Results are reported in Table 3, and scatterplots of key comparisons are illustrated in Fig. 7. The full correlation matrix is available in the Supplementary Materials.

**Table 3:** Selected Correlations Between Attentional Breadth and Eye Movement Behavior Measures

	Attentional Breadth Measures		AOSPAN Letters Correct	Memory Probe Score
	Kimchi-Palmer	Navon Score		
Fixation Metrics				
Mu	-0.084 [-0.261, 0.099] BF_10_ = 0.14	-0.011 [-0.192, 0.171] BF_10_ = 0.11	0.027 [-0.155, 0.208] BF_10_ = 0.12	-0.016 [-0.197, 0.166] BF_10_ = 0.12
Sigma	-0.018 [-0.199, 0.164] BF_10_ = 0.11	0.084 [-0.099, 0.262] BF_10_ = 0.17	0.026 [-0.156, 0.207] BF_10_ = 0.12	-0.063 [-0.242, 0.120] BF_10_ = 0.14
Tau	-0.157 [-0.329, 0.026] BF_10_ = 0.44	-0.199* [-0.367, -0.018] BF_10_ = 2.98	-0.089 [-0.266, 0.094] BF_10_ = 0.12	-0.254** [-0.417, -0.076] BF_10_ = 1.74
Saccade Amplitude	0.133 [-0.050, 0.307] BF_10_ = 0.29	0.167 [-0.015, 0.339] BF_10_ = 0.64	0.000 [-0.181, 0.182] BF_10_ = 0.11	-0.163 [-0.334, 0.019] BF_10_ = 0.25
Exploratory Breadth	0.161 [-0.021, 0.333] BF_10_ = 0.47	0.060 [-0.123, 0.239] BF_10_ = 0.13	-0.072 [-0.251, 0.111] BF_10_ = 0.13	0.111 [-0.072, 0.287] BF_10_ = 0.17
Scan Path Length	0.188* [0.006, 0.357] BF_10_ = 0.95	0.203* [0.022, 0.370] BF_10_ = 2.78	0.023 [-0.160, 0.203] BF_10_ = 0.11	0.076 [-0.107, 0.254] BF_10_ = 0.16
AOSPAN Letters Correct	0.246** [0.067, 0.409] BF_10_ = 3.53	0.147 [-0.035, 0.320] BF_10_ = 0.19	-	-
Memory Probe Score	0.057 [-0.126, 0.236] BF_10_ = 0.14	-0.031 [-0.211, 0.152] BF_10_ = 0.13	0.134 [-0.049, 0.308] BF_10_ = 0.31	-

*N* = 117. Spearman rank correlation coefficients used. Values in square brackets indicate 95% confidence intervals.

* *p* < 0.05, ** *p* < 0.01

There was very strong evidence in favor of a positive correlation between the two measures of attentional breadth (Kimchi-Palmer score and Navon score; *r*_s_ = 0.317, *p* < 0.001, BF_10_ = 36.51), indicating that attentional breadth preference was consistent across both tasks.

For the Kimchi-Palmer task, Bayes factors for significant correlations showed that there was almost equal support for the null and alternative hypotheses regarding association with scan path length (BF_10_ = 0.95), but there was moderate support for an association with AOSPAN Letters Correct (BF_10_ = 3.53). For the Navon difference score, associations with fixation tau (BF_10_ = 2.98) and scan path length (BF_10_ = 2.78) both received only anecdotal support. Finally, the relationship between memory probe score and fixation tau also received only anecdotal support (BF_10_ = 1.74).

#### Summary

Overall, task performance in this sample was typically consistent with patterns observed in previous research, such as showing a global preference in the Navon task, although working memory capacity of our participants was higher than a normative sample. The measurement reliability of key measures was excellent, providing a strong foundation from which to assess the relationships of interest between these measures. Spearman’s correlations showed (1) that a preference for a broader attentional breadth as assessed by the Navon task was associated with a more compact distribution of fixations during scene viewing, which is indicative of more consistent exploratory eye movement behavior, and (2) that a preference for a broad breadth of attention was consistently associated with the use of longer overall scan paths. However, these correlations received only anecdotal Bayes factor support, so they do not offer robust evidence of a meaningful relationship between these variables. Similarly, a robust relationship was detected between our measure of working memory capacity and only one measure of spatial deployment preference, indicating that in most instances there was not an association between spatial deployments and WMC.

## Experiment 2

In Experiment 1, we found only equivocal evidence for a relationship between preferences for different spatial deployments of attention, as well as an absence of any relationship with WMC for nearly all variables of interest. Experiment 2 therefore had two purposes: firstly, it sought to replicate the design of Experiment 1 to assess whether the same associations between different kinds of spatial deployment could be detected, and secondly, it tested the potential role of a different set of factors which may influence preferences for spatial attentional deployment: those related to beliefs about the world and enduring tendencies for interacting with it. Here, our measure of WMC (AOSPAN) was replaced by two questionnaires: NEO-FFI-3 (McCrae & Costa, 2007), a personality inventory, and the PI-99 (Clifton et al., 2019), a questionnaire which gauges primal beliefs about whether the world is good, safe, enticing, and alive. The behavioral tasks for Experiment 2 remained identical to those in Experiment 1 with one exception: the Kimchi-Palmer task was not included, as the positive correlation with Navon task performance indicated that it would be redundant to include two different measures of attentional breadth preference.

### Methods

#### Participants

Sample size was determined using the same approach and criteria as Experiment 1, resulting in a minimum sample size of 112 participants. Factoring in an extra 20% allowance for exclusions and technical issues, 134 participants were required; ultimately, 136 participants completed the study to keep the number of participants in each of four running orders (*n* = 34) equal; more information on running orders can be found in the ‘Materials and Procedure’ subsection. Eligibility criteria for participation were the same as Experiment 1, although participants who took part in Experiment 1 could not take part in Experiment 2.

For these 136 participants, mean age was 22.6 years (*SD* = 3.8 years).^4^ Thirty-six participants were male, 98 participants were female, one participant was non-binary, and one preferred not to disclose their gender. One hundred and twenty-eight participants reported being right-handed, six participants were left-handed, and two were ambidextrous. Twenty-nine participants reported wearing contact lenses, 28 participants reported wearing glasses, while the remaining 79 participants had normal vision. For country of birth, 51 participants reported an East Asian country (e.g. China, South Korea, Taiwan), 47 participants reported being born in Australia, ten reported a South Asian country (e.g. Bangladesh, India, Pakistan), ten reported a South East Asian country (e.g. Indonesia, Malaysia, Vietnam), nine reported a European country (e.g. Denmark, Germany, UK), while eight reported being born in another country (e.g. New Zealand, USA, Peru).^5^ 70 participants (51.5% of the sample) spoke English as a first language.

All participants provided written informed consent before participation. Participants were able to withdraw from participation at any time without penalty and could leave blank any questions they did not wish to answer in the final survey. The Australian National University’s Delegated Science and Medical Human Research Ethics Committee approved all ethical aspects of the experiment (Protocol 2022/119).

#### Materials and procedure

Participants were tested using the same laboratory setting and equipment setup described in Experiment 1. After informed consent was obtained, participants then completed four tasks: (1) a measure of attentional breadth preference (Navon task), (2) a measure of eye movement behavior preference (free viewing task with delayed recall), (3) the NEO-FFI-3 (McCrae & Costa, 2007), a 60-item personality inventory measuring five domains of personality (Neuroticism, Extraversion, Openness to Experience, Agreeableness and Conscientiousness), and (4) the Primals Inventory (PI-99; Clifton et al., 2019), a 99-item instrument that measures environment beliefs concerning the world’s overall character. For Experiment 2, four running orders were used, such that the ordering of tasks was varied while ensuring that the experimental tasks always occurred in the first half of the session and the questionnaires in the second half. This design choice minimized the impact of systematic error arising from a fixed running order upon task design, while also avoiding any impact of the participants’ experiences and reflections when completing the questionnaire measures upon experimental task performance (Laybourn et al., 2022; Schmader et al., 2008).

When learning about each of the experiment tasks, participants were given task instructions by the experimenter, and completed a practice block as described in the Methods section for Experiment 1. After completing the questionnaires, participants completed a short comprehension check activity where they provided definitions for three idioms taken from questions in the NEO-FFI-3 and PI-99 (e.g., the italicized idiom in ‘When I'm under a great deal of stress, sometimes I feel like I'm *going to pieces*.’), as well as a brief demographic survey. Finally, they were debriefed about the purpose of the experiment. Experiment sessions took approximately 60 min on average, and participants were compensated with either a cash payment ($20AUD) or research participation credit for relevant courses.

#### Experimental tasks and data preparation

For Experiment 2, the Navon and free viewing tasks were identical to those used in Experiment 1, as were data preparation procedures for these tasks. For the Navon task, this resulted in exclusion of a comparable number of each participant’s trials to Experiment 1 (*M* = 2.7%, *SD* = 0.8%). For the free viewing task, the entire datasets of participants with track loss rates of greater than 25% (*n* = 4) were excluded prior to event-level screening. Subsequently, 4.5% of fixation datapoints and 0.5% of saccade datapoints were excluded.

##### **NEO-FFI-3**

The NEO-FFI-3 (McCrae & Costa, 2007) consists of 60 items which measure the five domains of personality (Neuroticism, Extraversion, Openness to Experience, Agreeableness and Conscientiousness) by asking respondents to rate their level of agreement with a series of statements on a five-point scale: (1) Strongly Disagree, (2) Disagree, (3) Neutral, (4) Agree, (5) Strongly agree (scoring is inverted for reverse-coded items). Response scores for the 12 items in each domain are summed, such that a higher score for a domain is indicative of a higher level of the described trait. One attention check trial (e.g., ‘Please respond “Strongly Agree” to this question.’) was included in the NEO-FFI-3.

##### **Primals Inventory**

The Primals Inventory (PI-99; Clifton et al., 2019) consists of 99 items which measure a variety of environment beliefs concerning the world’s overall character (e.g., ‘On the whole, the world is a safe place.’). Respondents rate their level of agreement with a series of statements on a six-point scale: (1) Strongly Disagree, (2) Disagree, (3) Slightly Disagree, (4) Slightly Agree, (5) Agree, (6) Strongly Agree (scoring inverted for reverse-coded items). Response scores are averaged for four overarching beliefs of interest (that the world is Good, Safe, Enticing, and Alive), with a possible averaged score range of 1 to 6. Two attention check trials were included in the PI-99.

### Results

#### Overview

This Results section is divided into the following subsections: participant exclusions are summarized, experimental effects and task reliability are considered, and finally correlations between variables of interest are assessed.

#### Participant exclusions

Sample size was calculated at *n* = 134 (see Methods section), and 136 participants ultimately took part in the study. After 15 participant exclusions following screening for outliers, the final sample size was *n* = 121, which exceeded the required power for intended statistical analyses. Any participant who was excluded in a specific step of data screening was not counted again when they met an exclusion criterion in subsequent steps.

Datasets were initially screened for quality issues such as missing data and attention or comprehension issues for the questionnaire measures. As discussed above, four participants’ data were excluded because the rate of track loss for their eye tracking data exceeded 25% of trials. For the survey data, two participants’ data were excluded because they failed more than one of the three attention check questions, while a further four participants’ data were excluded because they failed to give a correct definition for more than one of the three idiom comprehension check questions. No participant skipped more than 3.7% of questions in the survey measures (*M* = 0.1%, *SD* = 0.5%), so no exclusions were performed on this basis and mode substitution was performed for missing items.

For the Navon task, the main dependent variable of interest was RT on correct-response trials, calculated after RT-based screening of each participant’s data (see Methods section). No participant had more than 10% of trials excluded by the RT-based screening procedure, so no exclusions were performed on this basis. A minimum criterion of 80% accuracy in all conditions was applied to ensure compliance with task instructions; no participants were excluded on this basis. Finally, univariate outlier screening was performed using a z-score criterion (± 3.29, *p* < 0.001) for RT metrics (Tabachnick & Fidell, 2013). One participant’s data were excluded on this basis.

For the free viewing task with delayed recall, data screening and fitting of ex-Gaussian distributions for fixation duration data were undertaken as described in the Methods section. One participant performed below 66.67% accuracy for the memory probe task, so this participant’s data were excluded on this basis. The same z-score criterion used for Navon outlier screening was applied to individual participants’ screened means for fixation duration, saccade amplitude, exploratory breadth (Euclidean distance of fixations from center), and scan path length; two participants’ data were excluded on this basis. Finally, the fitting of an ex-Gaussian distribution to fixation duration data failed to converge for one participant, whose data were excluded on this basis.

Overall, 15 participants’ data were excluded from further analysis, for a total sample of *n* = 121 in the final analysis. Analysis of the full sample prior to exclusions (see Supplementary Materials) indicated that all key conclusions remained unchanged between the screened and unscreened samples.

#### Distributions of experimental performance

Descriptive statistics for performance on tasks in Experiment 2 are reported in Table 1. Bayesian independent-samples t-tests performed in JASP 0.16 using default priors yielded at least moderate evidence against the hypothesis that performances differed across samples for Experiments 1 and 2 for all key experimental metrics except fixation *mu* (where evidence against this hypothesis was only anecdotal), and Navon global accuracy (where evidence in favor of this hypothesis was strong). This indicates that experimental performance was mostly consistent across the two samples. Running order had no impact upon either task performance or survey responses. Finally, as for Experiment 1, linear mixed-effects models analysis confirmed substantial differences in preference metrics between participants, as well as greater contributions to variance from participant-level effects relative to stimulus-level effects. These sample performance comparisons, running order analyses, and mixed-effects linear model analyses are all reported in full in the Supplementary Materials.

**Fig. 8:**
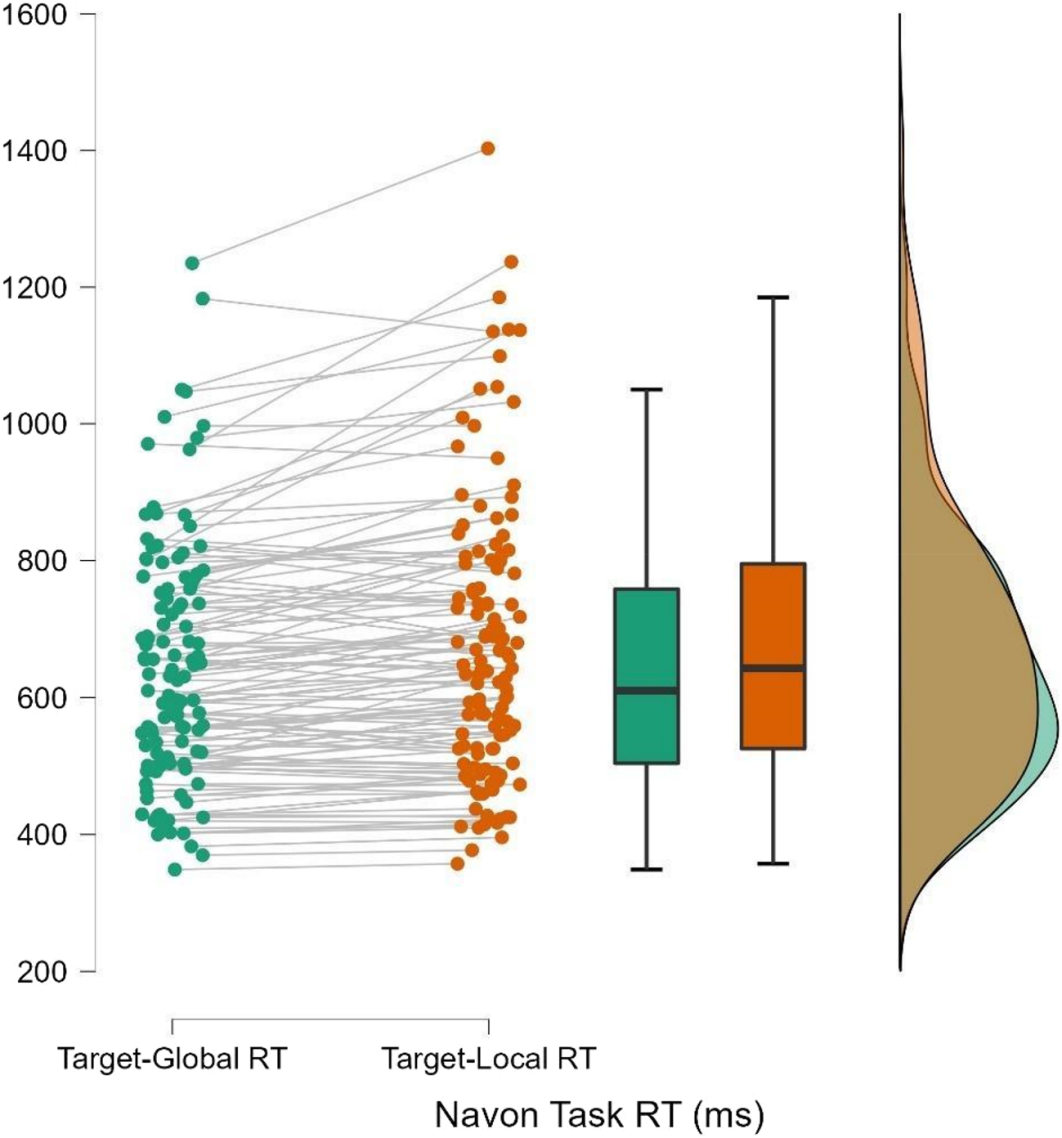
RT Performances by Participant for Navon Task in Experiment 2. *Note*. Performance slopes across Navon task conditions (target-global vs. target-local trials) are shown for each participant, as well as boxplots and distributions for sample performance in each condition. Performance in the target-global condition is shown in green, while performance in the target-local condition is shown in orange

RTs for the global and local trials of the Navon task were compared using a Wilcoxon signed-rank test.^6^ Despite considerable individual variance in attentional breadth preference (see Fig. 8), results indicated that participants’ RTs were faster for global trials than local trials; *z* = -5.074, *p* < 0.001, *r*_*rb*_^7^ = -0.534 (95% CI: [-0.665, -0.371]), replicating the global precedence effect observed in Experiment 1 and providing further converging evidence for the validity of our version of the Navon task.

#### Task reliability

As for Experiment 1, task reliability (i.e., the stability of participants’ individual performance levels across time on the experiment tasks) was examined to assess the extent to which the strength of any observed relationships between tasks were likely to be affected by this issue. Reliability for the difference score measure calculated for the Navon task, as well as the eye movement behavior metrics from the free viewing task, were estimated with the R package *splithalf* (Parsons, 2020) as per the procedure described in Experiment 1. This analysis indicated that all these metrics demonstrated good reliability (see Table 4) and were thus suitable for correlational analysis.

**Table 4:** Spearman-Brown Corrected Reliability Estimates for Selected Experimental Tasks in Experiment 2

Task	*r*_SB_^a^	95% CI	
		Lower	Upper
Navon Preference Score	0.75	0.56	0.86
Free Viewing Task			
Fixation Duration	0.98	0.97	0.98
Saccadic Amplitude	0.98	0.97	0.98
Exploratory Breadth	0.98	0.98	0.99
Scan Path Length	0.97	0.96	0.98

^a^ Spearman-Brown corrected reliability estimate

*N* = 121

#### Correlations

Finally, we addressed the two research questions that motivated this experiment (whether preferences for spatial-attentional deployments are related across different types of deployment, and whether any shared variance in these preferences is linked to personality and environment beliefs) with a correlational analysis. Shapiro–Wilk tests (reported in Supplementary Materials) indicated violations of the assumption of normality for some key variables, so we used a non-parametric test (Spearman’s rank correlation coefficients) to assess correlations between key variables of interest. Bayesian analysis was also conducted and interpreted as per Experiment 1. Correlations of theoretical interest are discussed below; the full correlation matrix is available in the Supplementary Materials.

As seen in Table 5, no significant correlations were found between Navon preference score and any of the eye movement behavior metrics.

**Table 5:** Correlations between Attentional Breadth and Eye Movement Behavior Measures

	Eye Movement Behavior Measures					
	Fixation Metrics			Saccade Amplitude (°)	Exploratory Breadth (px)	Scan Path Length (°)
	Mu	Sigma	Tau			
Navon Difference Score (ms)	-0.079 [-0.254, 0.101] BF_10_ = 0.16	0.008 [-0.171, 0.186] BF_10_ = 0.12	-0.008 [-0.187, 0.170] BF_10_ = 0.13	0.080 [-0.100, 0.254] BF_10_ = 0.12	-0.019 [-0.197, 0.160] BF_10_ = 0.11	0.088 [-0.091, 0.263] BF_10_ = 0.12

*N* = 121. Spearman rank correlation coefficients used. Values in square brackets indicate 95% confidence intervals. No correlation was significant at the *p* < 0.05 level

**Fig. 9:**
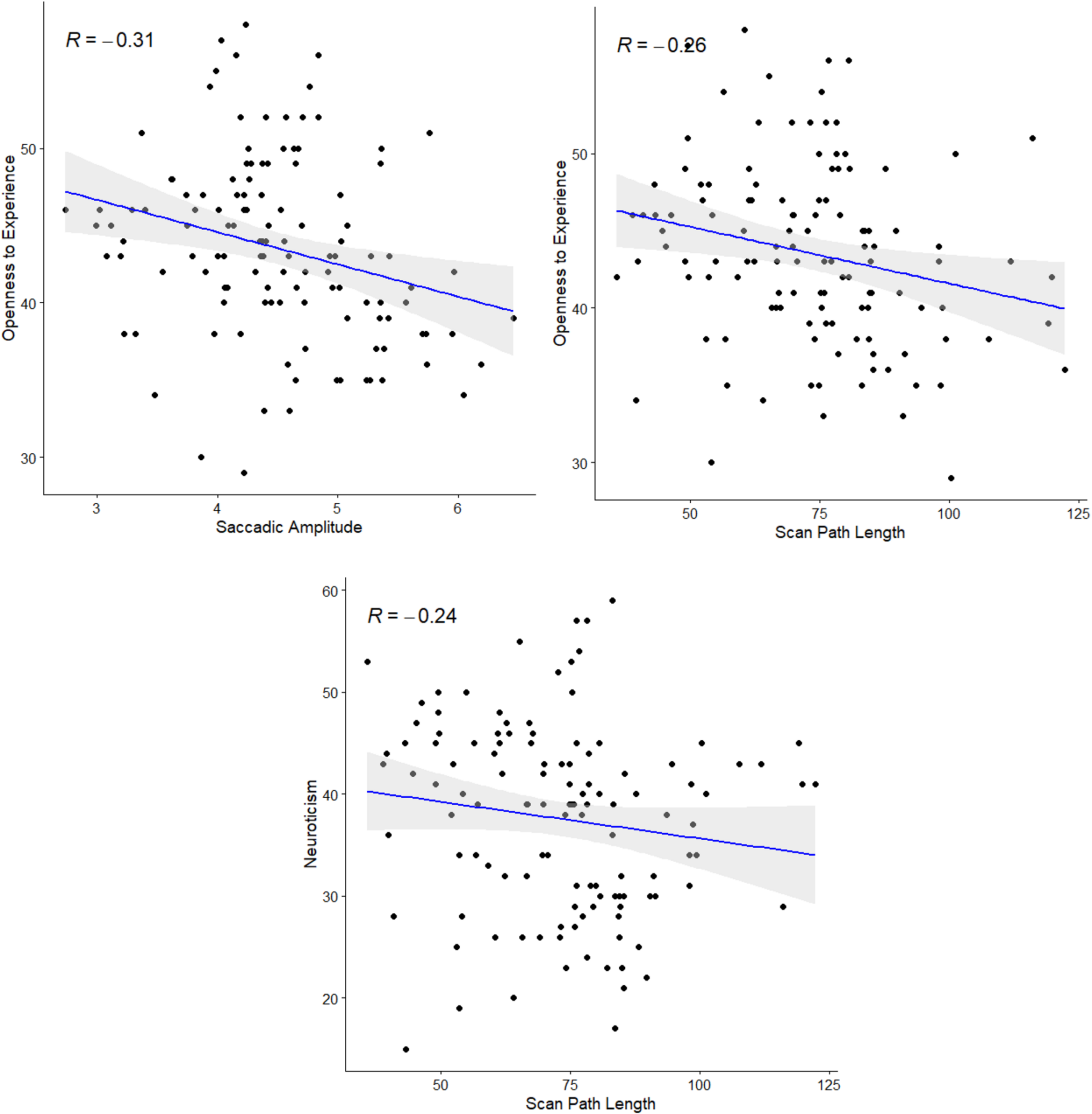
Scatterplots for Selected Correlations between Spatial Deployment and Personality Domain Measures. *Note*. Spearman correlations reported. Grey shaded area represents 95% confidence interval around regression line

As seen in Table 6, a small number of correlations were observed between measures of spatial deployment and the personality domains of Openness and Neuroticism. There was strong evidence in favor of the alternative hypothesis of a correlation between saccadic amplitude and Openness (*BF*_*10*_ = 10.35), and moderate evidence in favor of a correlation between scan path length and Openness (*BF*_*10*_ = 4.31). Conversely, there was moderate evidence *against* the hypothesis of a correlation between saccadic amplitude and Neuroticism (*BF*_*10*_ = 0.33). Scatterplots for these correlations can be seen in Fig. 9.

**Table 6:** Correlations between Spatial Deployment and Personality Domain Measures

Spatial Deployment Measures	Personality Domain Measures				
	Openness	Conscientiousness	Extraversion	Agreeableness	Neuroticism
Navon Difference Score (ms)	-0.177 [-0.345, 0.002] BF_10_ = 0.47	0.026 [-0.153, 0.204] BF_10_ = 0.12	-0.133 [-0.304, 0.047] BF_10_ = 0.20	-0.162 [-0.331, 0.072] BF_10_ = 0.30	-0.073 [-0.248, 0.107] BF_10_ = 0.15
Fixation Duration (mu)	0.036 [-0.143, 0.214] BF_10_ = 0.14	0.003 [-0.175, 0.182] BF_10_ = 0.11	0.126 [-0.053, 0.298] BF_10_ = 0.51	0.053 [-0.126, 0.230] BF_10_ = 0.12	-0.061 [-0.237, 0.118] BF_10_ = 0.11
Fixation Duration (sigma)	0.024 [-0.155, 0.202] BF_10_ = 0.12	-0.008 [-0.186, 0.171] BF_10_ = 0.12	0.120 [-0.059, 0.293] BF_10_ = 0.28	-0.002 [-0.180, 0.177] BF_10_ = 0.11	-0.048 [-0.224, 0.132] BF_10_ = 0.12
Fixation Duration (tau)	0.117 [-0.063, 0.289] BF_10_ = 0.19	-0.098 [-0.272, 0.082] BF_10_ = 0.15	0.014 [-0.164, 0.192] BF_10_ = 0.12	-0.022 [-0.200, 0.157] BF_10_ = 0.11	0.123 [-0.057, 0.295] BF_10_ = 0.14
Saccadic Amplitude (°)	-0.307*** **[-0.460, -0.136]** BF_10_ = 10.35	0.034 [-0.145, 0.211] BF_10_ = 0.13	-0.042 [-0.218, 0.138] BF_10_ = 0.11	-0.074 [-0.249, 0.106] BF_10_ = 0.13	-0.171 [-0.339, 0.008] BF_10_ = 0.35
Exploratory Breadth (px)	-0.132 [-0.304, 0.047] BF_10_ = 0.15	0.029 [-0.151, 0.206] BF_10_ = 0.11	0.067 [-0.113, 0.242] BF_10_ = 0.15	0.003 [-0.176, 0.181] BF_10_ = 0.12	-0.132 [-0.303, 0.048] BF_10_ = 0.19
Scan Path Length (°)	-0.262** **[-0.421, -0.087]** BF_10_ = 4.31	0.107 [-0.073, 0.280] BF_10_ = 0.20	-0.076 [-0.251, 0.104] BF_10_ = 0.13	-0.027 [0.205, 0.152] BF_10_ = 0.11	-0.238** **[-0.399, -0.062]** BF_10_ = 0.33

*N* = 121. Spearman rank correlation coefficients used. Values in square brackets indicate 95% confidence intervals. Correlations significant at the *p* < 0.05 level are bolded.

** *p* < 0.01, *** *p* < 0.001

No associations were found between any measure of spatial deployment and the environment beliefs measured by the PI-99 (see Supplementary Materials for correlation matrix).

## Summary

Task performance in Experiment 2 was consistent with that observed in both Experiment 1 and previous research. The measurement reliability of key measures was good, which permitted a correlational analysis of the relationships between measures of interest. Here, no relationship was found between attentional breadth and eye movement behavior messages, suggesting that the correlations observed in Experiment 1 may have been spurious. Regarding relationships between spatial deployment metrics and trait variables (personality and environment beliefs), higher levels of Openness to Experience were robustly associated with the use of smaller saccades and shorter overall scan paths. Conversely, no relationships were observed between spatial deployment metrics and environment beliefs.

## General discussion

Humans can regulate their spatial attention in multiple different ways, including changing the size of the attended region (i.e., attentional breadth) and changing the spatial extent of the area over which eye movements are made. The primary aim of this exploratory study was to assess whether there were consistencies in how broadly versus narrowly individuals preferred to apply their attention across these two different kinds of spatial-attentional deployment, in contexts where task constraints on these behaviors were minimal. To do this, we used two tasks that gauged attentional breadth preference (a Kimchi-Palmer task and an undirected Navon task) and one task that gauged eye movement behavior (a scene viewing task). Results from Experiment 1 showed that preference for a broader attentional breadth as assessed by the Navon task was associated with a more compact distribution of fixations during scene viewing (indicative of more regular patterns of exploratory eye movement behavior). Additionally, preference for a broad breadth of attention as assessed by both tasks was consistently associated with the use of longer overall scan paths. However, Bayes factor support for these correlations was only anecdotal, suggesting that these correlational relationships are not robust enough to confidently conclude a meaningful association between these variables. Indeed, in Experiment 2 (where only the undirected Navon task was used to measure attentional breadth preference), no significant relationships were observed between attentional breadth preference and eye movement behavior, suggesting that tendencies for the deployment of spatial attention may not be reliably associated across deployment types.

The second aim of this study was to assess the potential roles of other factors in determining preferences for the deployment of spatial attention. Given the role of WMC in the *ability* to regulate both attentional breadth and eye movement behavior, in Experiment 1 we investigated whether WMC explained any shared variance in individual *preferences* for these kinds of spatial deployment. Participants’ WMC scores only correlated with a single measure of spatial deployment preference, suggesting that WMC does not play an important role in determining these preferences. In Experiment 2 we investigated potential relationships between aspects of participants’ worldviews and their preferences of deployments of spatial attention. We found small but robust negative associations between the personality trait of Openness and two eye movement behavior variables (mean saccadic amplitude and mean scan path length), counterintuitively suggesting that openness to experience correlates with *less* exploratory behavior.

The following sections will consider the implications of the study’s two key findings: firstly, the equivocal nature of the evidence about whether some aspects of eye movement behavior are related to preferences for attentional breadth, and secondly, the roles of other factors in determining preferences for the spatial deployment of attention.

## Relationships between preferences for spatial deployment of attention

Given the equivocal and inconsistent relationships observed in this study, we favor the interpretation that preferences for the deployment of spatial attention are not robustly related across different kinds of spatial deployment. We prefer this interpretation for two reasons: (1) the lack of an overarching pattern of association between the types of spatial deployment which made sense at a theoretical level, and (2) the equivocal nature of the correlations observed in Experiment 1 as well as their failure to replicate in Experiment 2. Firstly, even if the relationships between some aspects of eye movement behavior and attentional breadth preference observed in Experiment 1 were robust, it is surprising that these relationships were not systematically present for other aspects (i.e., saccade amplitude, exploratory breadth). For instance, if overarching preferences remained consistent across different types of spatial deployment, preference for a larger attentional breadth should lead an individual to use larger saccades, simply because their eyes would need to move further to access information that falls outside the bounds of the attended region. However, these systematic findings did not eventuate, suggesting that there is no core preference which determines how different kinds of spatial attention are applied. Secondly, there were issues with the strength and replicability of the relationships observed in this study. Bayesian analysis of the correlational relationships in Experiment 1 revealed that no relationship of interest received better than ‘anecdotal’ support (Andraszewicz et al., 2014), even though the excellent reliability of our measurements means that the strength of the observed relationships is unlikely to have been attenuated by error variance. The failure to replicate any relationship between different deployments of attention in Experiment 2 lends further credence to the idea that these preferences are truly unrelated.

Overall, the findings from this study do not support the idea that individual spatial-attentional deployment preferences are consistent across different types of deployment. Indeed, if the question is whether there are *robust* relationships between attentional breadth and exploratory eye movement behavior, then the results obtained in this study indicate an answer of ‘no’. Where there *was* resounding consistency across both experiments was within individuals’ eye movement behavior across scenes. While these did not appear to relate strongly and consistently to behavioral tendencies on the attentional breadth task, it indicates that there are substantial and robust individual differences in eye movement behavior (see also Wyche et al., 2023).

Another implication of the present results is apparent support for the distinction between preference and ability. Although preference and ability metrics were not directly compared here, the pattern of results – where there were at best modest relationships between attentional breadth and eye movement preferences – diverges from that found previously with ability measures of attentional breadth and eye movements (Weber et al., 2000). This suggests that it is important to consider whether specific tasks operationalize attentional preferences versus ability when designing experimental research. Conversely, an open question is whether ability *influences* preferences about how visual information is acquired. For instance, it is conceptually logical that individuals with higher working memory capacity may be more effective at processing larger amounts of visual information simultaneously. Consequently, the attentional preferences of individuals with varying levels of working memory capacity may be constrained by this factor: individuals with lower working memory capacities may need to shift the focus of attention more frequently to collect an equivalent level of information to that which individuals with higher working memory capacities could acquire from a single fixation. Such an interpretation is supported by the findings of Luke et al. (2018) where higher WMC was linked to longer fixations in a scene-viewing task, but this finding was not replicated in the present study. Overall, more work is needed to determine whether information processing ability plays a causal role in the formation of spatial-attentional preferences.

## Factors associated with preferences for spatial-attentional deployment

A secondary purpose of this experiment was to identify factors associated with how individuals tend to deploy spatial attention. Across two experiments, we tested two possible sets of factors, finding a nearly total absence of relationship with WMC in Experiment 1, and some modest but robust associations with the personality trait of Openness in Experiment 2. These results and their implications are considered below.

## WMC

In Experiment 1, we tested WMC as a candidate which could account for variance in spatial-attentional deployment. In previous work both we (Wyche et al., 2023) and other authors (Cronin et al., 2020a, b) have shown that working memory *load* alters some eye movement behaviors, although evidence about its influence on attentional breadth is mixed (Ahmed & de Fockert, 2012a, 2012b; Caparos & Linnell, 2010; Hoar & Linnell, 2013; Linnell & Caparos, 2011; Yao et al., 2020). Additionally, WMC is known to affect the *ability* to regulate both attentional breadth (Goodhew, 2020b; Kreitz et al., 2015) and eye movement behavior (Hayes & Henderson, 2017; Luke et al., 2018). Evidence about its role in eye movement behavior *preferences* is mixed: one study supports an association between preference behavior and WMC (Luke et al., 2018), while another did not replicate this finding (Loh et al., 2022). We therefore included a measure of working memory capacity to assess whether it accounted for any variance in preference strategies across attentional breadth and eye movement behavior. We found that WMC was associated with only a single metric of spatial deployment preference (the Kimchi-Palmer task), such that higher WMC was linked to a preference for a broader breadth of attention.

One possible explanation for our findings is that unusually strong performance in our sample on the WMC task (AOSPAN) constrained our ability to observe relationships with the other tasks. Participants’ scores on AOSPAN were significantly better than benchmark levels indicated by normative data (Redick et al., 2012), and an examination of the spread of AOSPAN performance revealed a clustering of scores at or close to ceiling-level performance. The reason for this unusually high sample performance is not immediately clear; our implementation of AOSPAN conformed to the standardized task design, and an experimenter supervised the administration of the task to ensure that participants did not use any alternative strategies such as repeating the letters to be recalled out loud. Regardless, this issue with ceiling effects in WMC measurement is not unique to our study, and it has been suggested that problems with capturing a range of differences in WMC for higher-ability samples such as university students may affect the ability to detect relationships between working memory functioning and spatial deployments of attention (Draheim et al., 2018; Loh et al., 2022). Before ruling out a relationship between WMC and preferences for the spatial deployment of attention, future work will need to test WMC in the limit to obtain a sensitive individual-differences measure which is not constrained by sample ceiling effects.

However, it remains possible that WMC is genuinely unrelated to preferences for the deployment of spatial attention, or that only specific aspects of working memory functioning relate to eye movement behavior. There has been some debate about how WMC as measured by complex span tasks like AOSPAN relates to attentional functioning: some authors have linked WMC to the functioning of selective attention (see Vandierendonck, 2014 for a review), while other research evidence links it more specifically to attentional control in goal-directed behavior (see Chow & Conway, 2015 for a review). Therefore, one account which could reconcile the findings of previous research and the present study is that WMC plays a role in an individual’s *ability* to strategically adapt their use of spatial attention in response to task demands, but it does not affect individual *preferences* for particular kinds of spatial-attentional deployment when no such strategic demands are being imposed. It is simultaneously possible that only specific aspects of visuospatial working memory functioning which are well-understood to be involved in eye movement behavior (e.g., oculomotor control, presaccadic encoding into visual working memory, perceptual continuity across saccades, and post-saccadic gaze correction; van der Stigchel & Hollingworth, 2018) are implicated in behavioral preferences, rather than the domain-general conception of WMC which is gauged by AOSPAN. If these accounts are substantiated, research into other trait factors which can account for individual variance in preference for spatial deployments of attention might be more fruitful avenues of investigation.

## Personality and world beliefs

In Experiment 2, we responded to the lack of association between preferences for the deployment of spatial attention and WMC by investigating the possibility of relationships with altogether different kinds of trait variables: *personality* and *environment beliefs*. Personality has previously shown some associations with both attentional breadth preference (Swift et al., 2020; Wilson et al., 2016) and eye movement behavior (Hoppe et al., 2018; Rauthmann et al., 2012), while environment beliefs are the focus of a relatively new literature and have not previously been studied in relation to deployments of spatial attention. Ultimately, we found that higher levels of trait Openness to Experience were modestly but robustly associated with the use of shorter saccades and shorter overall scan paths on each trial. We found no relationship between attentional breadth preferences and personality traits, nor did we find any relationships between environment beliefs and preferences for the spatial deployment of attention.

These findings do not replicate some past observations about the relationship between personality and spatial attention, but they are conceptually convergent with other aspects of past findings. Firstly, it is notable that attentional breadth preference was not reliably related to any personality trait in our study,^8^ despite measures of attentional breadth in previous studies (an inhibition of return paradigm in Wilson et al., 2016, and a Kimchi-Palmer paradigm in Swift et al., 2020) conforming to the definition of preference measures that we provide in the Introduction. Secondly, with respect to eye movement behaviors, we did not replicate the findings of Rauthmann et al. (2012) regarding a relationship between personality variables and fixation duration, and in contrast to Hoppe et al. (2018), Openness was the *only* variable we found to be related to eye movement behavior preferences! However, if we conceptualize sustained engagement and active exploration as opposite ends of a spectrum of tendency to explore available information (Hills et al., 2015), there is some conceptual convergence between our findings and those of Rauthmann et al. (2012). The finding that those higher on Openness preferred longer fixation durations in Rauthmann et al. (2012), as well as shorter saccades and scan paths in our Experiment 2, may indicate a greater orientation towards sustained *engagement* in individuals high on Openness. Facets of the construct of Openness such as aesthetic sensitivity and intellectual curiosity may plausibly promote a higher level of absorbed engagement of attention when examining visual stimuli, and some research has indicated that eye movements can be used as an index of curiosity (Baranes et al., 2015; Hoppe et al., 2015). Alternatively, higher levels of Openness to Experience may predict a preference for a memorization strategy on the scene viewing task whereby a smaller subregion of each scene is encoded to memory at a higher level of detail. Therefore, an interesting direction for future research would be investigating whether specific *facets* of Openness have stronger associations with eye movement behavior than the relatively modest relationships observed in this study.

It is also necessary to draw some methodological distinctions between the eye movement metrics in the present study, and those by Rauthmann et al. (2012) and Hoppe et al. (2018). Rauthmann et al. (2012) used computer-generated animations as stimuli, while the experimental design of Hoppe et al. (2018) involved participants wearing a head-mounted eye tracker during completion of an everyday task, both clear points of difference from the present study, which used real pictures of nature scenes in a static, lab-based setting. These differences offer a potential alternate explanation for the failure to replicate some findings from these other studies, because it is known that patterns of eye movement behavior during viewing of videos (Smith & Mital, 2013) and movement in real-world environments (Foulsham & Kingstone, 2017; Foulsham et al., 2011) differ substantially from those which are observed during viewing of still images. Further research comparing eye movement behavior preferences across different contexts is needed: ideally, a single-study design using eye tracking for still images, videos and real-world settings could conclusively determine whether eye movement behavior in different contexts is genuinely associated with disparate personality traits.

## Conclusion

In conclusion, this study investigated whether there are overarching consistencies in the ways in which individuals tend to deploy spatial attention. It also sought to identify other factors which might be associated with individual preferences for the deployment of spatial attention, specifically WMC and worldviews. This study did not find robust evidence for an association between how individuals prefer to deploy attentional breadth and eye movements: some equivocal correlations detected in Experiment 1 did not receive Bayes factor support, and they were not replicated in Experiment 2. Further, while Experiment 1 did not find that WMC played a significant role in determining preferences for how spatial attention is deployed, Experiment 2 found that Openness to Experience was associated with eye movement behavior that reflected more sustained *engagement* with the visual stimuli.

We wish to emphasise two important caveats regarding the results of this study. Firstly, interpretation of these findings is contingent upon whether they accurately reflect the nature of the underlying relationships being studied. One of the challenges of exploratory research is that there is no yardstick against which to evaluate these findings: although we have made efforts to ensure that our data is of a high quality and our interpretations are well-founded, it is difficult to know whether the relationships we have observed represent false positives, or whether those we have failed to find evidence for are in fact false negatives. This point is especially pressing given the weak relationships between different measures of attentional preference from Experiment 1, which failed to replicate in Experiment 2. In light of these issues, a direct replication of this study’s findings would be an important prerequisite to future research regarding attentional preferences, as it would provide confidence that the relationships (or lack thereof) which we have observed are a genuine reflection of the population.

 Important to acknowledge that only a handful of correlations among the many reported in this study were robustly significant. However, we draw attention once more to the exploratory nature of this study: these findings are also valuable for the evidence they provide about which associations were *not* significant. Across two experiments, we have seen that preferences for different deployments of spatial attention are not reliably related. Further, in light of previous research findings about the effect of working memory load upon deployments of spatial attention (Wyche et al., 2023), it is intriguing to note that preference behavior in this domain is largely unassociated with working memory *capacity*. Having undertaken this foundational exploratory work, future research would ideally replicate the findings we have highlighted before testing whether trait factors that are predictive of attentional breadth preferences such as neurodivergence or culture are also associated with exploratory breadth preferences (Caparos et al., 2013; Koldewyn et al., 2013), as well as whether different facets of Openness to Experience such as aesthetic sensitivity and intellectual curiosity have superior predictive value for preferences in eye movement behavior.

## Supplementary information

Below is the link to the electronic supplementary material.

Supplementary file1 (PDF 338 KB)

## Data Availability

The data and materials for all experiments are available at: https://osf.io/aqdb4/.

## Footnotes

1 Rank-biserial correlation, used as a measure of effect size for non-parametric paired-samples comparisons (Kerby, 2014).

2 We did not calculate reliability for the Kimchi-Palmer because variation of stimulus layouts and density across trials leads to sample-level changes in the pattern of responses (Kimchi & Palmer, 1982). Therefore, calculation of a reliability measure for this task is not possible: the trials are not directly comparable and an internally consistent pattern of responses is not necessarily expected.

3 95% confidence intervals for Cronbach’s alpha were calculated using the R package *alphaci* (Moss, 2022) with default settings and 1000 bootstraps.

4 One participant did not provide their age.

5 One participant did not provide their country of birth.

6 A non-parametric test was used as these RT scores were not normally distributed; see Supplementary Materials.

7 Rank-biserial correlation, used as a measure of effect size for non-parametric paired-samples comparisons (Kerby, 2014).

8 While our measure of attentional breadth preference (Navon preference score) nearly attained frequentist significance for a correlation with Openness (*r*_*S*_ = -0.177, *p* = .052), there was anecdotal evidence in favor of the null hypothesis that these variables were *unrelated* (*BF*_10_ = 0.450). This correlation is in the opposite direction to the relationship reported in Wilson et al. (2016), but concords with Swift et al.’s (2020) finding that higher levels of Openness predict more efficient ability to *contract* the breadth of attention.
