# Supplementary material for: Openness to experience predicts eye movement behavior during scene viewing
Source: Atten Percept Psychophys. 2024 Aug 12;86(7):2386–411. doi: 10.3758/s13414-024-02937-z (PMC11480192; doi:10.3758/s13414-024-02937-z)
Supplement: Supplementary file 1 — Supplementary file1 (PDF 338 KB) [file 13414_2024_2937_MOESM1_ESM.pdf]

## Supplementary Materials

## Part 1: Additional Analyses for Experiments 1 and 2

### *Shapiro-Wilk Tests*

**Table A1**

*Shapiro-Wilk Tests for Key Variables in Experiments 1 and 2*

| Variable | Experiment 1 | | Experiment 2 | |
|---|---|---|---|---|
| | W | *p* | W | *p* |
| Attentional Breadth | | | | |
| Kimchi-Palmer Score | .887 | < .001 | - | - |
| Navon Global RT | .927 | < .001 | .952 | < .001 |
| Navon Local RT | .933 | < .001 | .936 | < .001 |
| Navon Score | .897 | < .001 | .886 | < .001 |
| Eye Movement Behaviour | | | | |
| Saccade Amplitude Mean | .987 | .328 | .990 | .514 |
| Exploratory Breadth Mean | .983 | .132 | .993 | .833 |
| Scan Path Length Mean | .991 | .649 | .981 | .080 |
| Fixation Mu | .993 | .811 | .983 | .131 |
| Fixation Sigma | .914 | < .001 | .961 | .001 |
| Fixation Tau | .942 | < .001 | .956 | < .001 |
| Memory Probe Score | .860 | < .001 | .667 | < .001 |
| AOSPAN Letters Correct | .835 | < .001 | - | - |
| NEO-FFI | | | | |
| Neuroticism | - | - | .985 | .188 |

| | | | | |
|---|---|---|---|---|
| Extraversion | - | - | .982 | .098 |
| Openness | - | - | .993 | .790 |
| Agreeableness | - | - | .988 | .339 |
| Conscientiousness | - | - | .959 | .001 |
| PI-99 | | | | |
| Good | - | - | .993 | .826 |
| Safe | - | - | .991 | .650 |
| Enticing | - | - | .970 | .008 |
| Alive | - | - | .982 | .100 |

*Note.* The Shapiro-Wilk test assesses whether variables are normally distributed. $p < .05$ indicates support for the alternative hypothesis that a variable is *not* normally distributed.

***Comparison of Task Performance in Experiments 1 and 2***

**Table A2**

*Comparison of Task Performance in Experiment 1 and 2*

| Variable | Exp 1 | | Exp 2 | | $BF_{10}$ |
|---|---|---|---|---|---|
| | *M* | *SD* | *M* | *SD* | |
| Navon Task | | | | | |
| Global Accuracy (%) | 98.10 | 2.12 | 98.86 | 1.40 | 20.457 |
| Local Accuracy (%) | 97.59 | 2.85 | 98.00 | 2.39 | 0.275 |
| Global RT (ms) | 631.76 | 139.45 | 643.55 | 175.62 | 0.166 |
| Local RT (ms) | 678.48 | 176.43 | 678.00 | 205.67 | 0.142 |
| Preference Score (ms) | 46.72 | 83.38 | 34.45 | 66.20 | 0.300 |
| Free Viewing Task | | | | | |
| Memory Test (%) | 94.80 | 5.30 | 95.20 | 5.00 | 0.165 |
| Fixation Duration (mu) | 160.46 | 23.08 | 155.87 | 24.66 | 0.398 |
| Fixation Duration (sigma) | 57.40 | 14.54 | 56.15 | 10.64 | 0.187 |
| Fixation Duration (tau) | 143.55 | 40.00 | 144.49 | 38.48 | 0.144 |
| Saccadic Amplitude (°) | 4.42 | 0.82 | 4.49 | 0.75 | 0.181 |
| Exploratory Breadth (pixels) | 248.98 | 36.09 | 255.84 | 40.88 | 0.344 |
| Scan Path Length (°) | 72.50 | 17.24 | 73.32 | 18.15 | 0.151 |

*Note.* For Experiment 1, *N* = 117. For Experiment 2, *N* = 121. Bayes factors are for independent-samples t-tests performed in JASP 0.16 using default priors to assess evidence for the hypothesis that performances differed across samples for Experiments 1 and 2.

***Experiment 2 Running Order Analysis***

**Table A3**

*Effects of Task Running Order on Performance in Experiment 2*

| Variable | t | df | *p* | $BF_{10}$ |
|---|---|---|---|---|
| Navon Global RT | -0.862 | 119 | 0.39 | 0.27 |
| Navon Local RT | -0.984 | 119 | 0.327 | 0.30 |
| Navon Score | -0.766 | 119 | 0.445 | 0.25 |
| Memory Probe Accuracy | 0.458 | 119 | 0.648 | 0.21 |
| Saccade Amplitude Mean | 0.65 | 119 | 0.517 | 0.24 |
| Exploratory Breadth Mean | 0.776 | 119 | 0.439 | 0.25 |
| Scan Path Length Mean | 0.055 | 119 | 0.957 | 0.19 |
| Fixation Mu | 0.144 | 119 | 0.886 | 0.20 |
| Fixation Sigma | 0.241 | 119 | 0.81 | 0.20 |
| Fixation Tau | -0.12 | 119 | 0.904 | 0.20 |

*Note*. $N = 121$. Independent-samples t-tests were performed in JASP 0.16 to assess whether performance differed significantly across task running orders (indicated by $p < .05$).

**Table A4**

*Effects of Questionnaire Running Order on Performance in Experiment 2*

| Variable | t | df | *p* | $BF_{10}$ |
|---|---|---|---|---|
| NEO-FFI Openness | 0.079 | 119 | 0.937 | 0.195 |
| NEO-FFI Conscientiousness | -1.487 | 119 | 0.14 | 0.525 |
| NEO-FFI Extraversion | -1.376 | 119 | 0.171 | 0.456 |
| NEO-FFI Agreeableness | -0.18 | 119 | 0.857 | 0.197 |
| NEO-FFI Neuroticism | 0.406 | 119 | 0.685 | 0.209 |
| PI-99 Good | -2.062 | 119 | 0.041 | 1.302 |
| PI-99 Safe | -1.336 | 119 | 0.184 | 0.434 |
| PI-99 Enticing | -1.859 | 119 | 0.065 | 0.915 |
| PI-99 Alive | -1.506 | 119 | 0.135 | 0.538 |

*Note*. *N* = 121. Independent-samples t-tests were performed in JASP 0.16 to assess whether performance differed significantly across task running orders (indicated by $p < .05$).

### *Linear Mixed Model: Variance Components*

Before a correlational analysis of measures of interest, we sought to determine the proportion of variance in our experimental tasks which was accounted for by individual differences in participant performance. Therefore, we fitted linear mixed-effects models to event-level data for the following experimental variables of interest: (a) fixation durations, (b) saccadic amplitudes, (c) exploratory breadth, and trial-level data for two further variables: (d) Navon RTs, and (e) scan path lengths. We were interested in the variance contributed not only by participants, but also by experimental stimuli, due to the possibility that eye movement behaviour was affected primarily by stimulus features such as contours. Therefore, to identify how much variance these components contributed toward task performance, we entered random effects only in these models, such that intercepts were entered for both participants and stimuli. Analyses were preformed using the GAMLj package for jamovi 2.3.16 (Gallucci, 2022). Intra-class correlations (ICCs) for these random effects are reported for both Experiments 1 and 2 in Table A5, indicating that for all variables, clustering from each individual participant exceeded clustering from each stimulus type.

**Table A5**

*Intra-Class Correlations for Random Effects of Participant and Stimulus upon Experimental Variables*

| Variable | Experiment 1 | | Experiment 2 | |
|---|---|---|---|---|
| | Participants | Stimuli | Participants | Stimuli |
| Navon RT (ms) | 0.249 | 0.018 | 0.216 | 0.003 |
| Free Viewing Task | | | | |
| Fixation Duration (ms) | 0.08 | 0.003 | 0.065 | 0.003 |

| | | | | |
|---|---|---|---|---|
| Saccade Amplitude (°) | 0.061 | 0.014 | 0.049 | 0.01 |
| Exploratory Breadth (px) | 0.058 | 0.031 | 0.072 | 0.026 |
| Scan Path Length (°) | 0.385 | 0.127 | .402 | 0.092 |

***Full Correlation Tables***

## Table A6

*Correlations between Measures of Interest in Experiment 1*

| | Kimchi-Palmer Score | Navon Score | AOSPAN Letters Correct | Memory Probe Accuracy | Saccade Amplitude Mean | Exploratory Breadth Mean | Scan Path Mean | Fixation Mu | Fixation Sigma |
|---|---|---|---|---|---|---|---|---|---|
| Navon Score | .317*** [.143, .471] $BF_{10}$ = 36.51 | - | | | | | | | |
| AOSPAN Letters Correct | .246** [.067, .409] $BF_{10}$ = 3.53 | .147 [-.035, .320] $BF_{10}$ = 0.19 | - | | | | | | |
| Memory Probe Accuracy | .057 [-.126, .236] $BF_{10}$ = 0.14 | -.031 [-.211, .152] $BF_{10}$ = 0.13 | .134 [-.049, .308] $BF_{10}$ = 0.31 | - | | | | | |
| Saccade Amplitude Mean | .133 [-.050, .307] $BF_{10}$ = 0.29 | .167 [-.015, .339] $BF_{10}$ = 0.64 | .000 [-.181, .182] $BF_{10}$ = 0.11 | -.163 [-.334, .019] $BF_{10}$ = 0.25 | - | | | | |
| Exploratory Breadth Mean | .161 [-.021, .333] $BF_{10}$ = 0.47 | .060 [-.123, .239] $BF_{10}$ = 0.13 | -.072 [-.251, .111] $BF_{10}$ = 0.13 | .111 [-.072, .287] $BF_{10}$ = 0.17 | .540*** [.398, .657] $BF_{10}$ =1.6e6 | - | | | |
| Scan Path Length Mean | .188* [.006, .357] $BF_{10}$ = 0.95 | .203* [.022, .370] $BF_{10}$ = 2.78 | .023 [-.160, .203] $BF_{10}$ = 0.11 | .076 [-.107, .254] $BF_{10}$ = 0.16 | .671*** [.558, .760] $BF_{10}$ =9.9e10 | .632*** [.509, .730] $BF_{10}$ = 3.8e9 | - | | |
| Fixation Mu | -.084 [-.261, .099] $BF_{10}$ = 0.14 | -.011 [-.192, .171] $BF_{10}$ = 0.11 | .027 [-.155, .208] $BF_{10}$ = 0.12 | -.016 [-.197, .166] $BF_{10}$ = 0.12 | .214* [.034, .381] $BF_{10}$ =13.11 | -.012 [-.194, .169] $BF_{10}$ = 0.11 | -.142 [-.315, .041] $BF_{10}$ = 0.19 | - | |
| Fixation Sigma | -.018 [-.199, .164] $BF_{10}$ = 0.11 | .084 [-.099, .262] $BF_{10}$ = 0.17 | .026 [-.156, .207] $BF_{10}$ = 0.13 | -.063 [-.242, .120] $BF_{10}$ = 0.14 | .218* [.038, .384] $BF_{10}$ = 5.12 | -.080 [-.258, .103] $BF_{10}$ = 0.16 | -.178 [-.349, .003] $BF_{10}$ = 0.61 | .694*** [.586, .778] $BF_{10}$ =9.6e13 | - |

| | | | | | | | | | |
|---|---|---|---|---|---|---|---|---|---|
| Fixation Tau | -.157<br>[-.329, .026]<br>$BF_{10}$ = 0.44 | -.199*<br>[-.367, -.018]<br>$BF_{10}$ = 2.98 | -.089<br>[-.266, .094]<br>$BF_{10}$ = 0.12 | -.254**<br>[-.417, -.076]<br>$BF_{10}$ = 1.74 | .005<br>[-.177, .186]<br>$BF_{10}$ = 0.17 | -.222*<br>[-.388, -.043]<br>$BF_{10}$ = 1.62 | -.609***<br>[-.712, -.481]<br>$BF_{10}$ =9.3e9 | .108<br>[-.075, .284]<br>$BF_{10}$ = 0.19 | .173<br>[-.009, .343]<br>$BF_{10}$ = 0.58 |

*Note.* $N$ = 117. Spearman rank correlation coefficients used. Values in square brackets indicate 95% confidence intervals. Bayes factors are calculated using the approach described in van Doorn et al. (2020), which permits Bayesian inference for Spearman rank correlation coefficients.

* $p < .05$, ** $p < .01$, *** $p < .001$

**Table A7**

*Correlations between Experimental Task Performance in Experiment 2*

| | Navon Score | Memory Probe Accuracy | Saccade Amplitude Mean | Exploratory Breadth Mean | Scan Path Mean | Fixation Mu | Fixation Sigma |
|---|---|---|---|---|---|---|---|
| Memory Probe Accuracy | -.007 [-.185, .172] $BF_{10}$ = 0.11 | - | | | | | |
| Saccade Amplitude Mean | .080 [-.100, .254] $BF_{10}$ = 0.12 | .143 [-.037, .313] $BF_{10}$ = 0.35 | - | | | | |
| Exploratory Breadth Mean | -.019 [-.197, .160] $BF_{10}$ = 0.11 | .340*** [.172, .489] $BF_{10}$ =143.25 | .506*** [.360, .628] $BF_{10}$ =8.3e5 | - | | | |
| Scan Path Length Mean | .088 [-.091, .263] $BF_{10}$ = 0.12 | .320*** [.150, .472] $BF_{10}$ = 76.01 | .714*** [.614, .791] $BF_{10}$ = 6.9e15 | .526*** [.384, .644] $BF_{10}$ = 1.3e5 | - | | |
| Fixation Mu | -.079 [-.254, .101] $BF_{10}$ =0.16 | .090 [-.090, .264] $BF_{10}$ = 0.20 | .150 [-.030, .320] $BF_{10}$ = 0.30 | -.002 [-.180, .177] $BF_{10}$ = 0.11 | -.005 [-.183, .174] $BF_{10}$ = 0.12 | - | |
| Fixation Sigma | .008 [-.171, .186] $BF_{10}$ = 0.12 | -.004 [-.183, .174] $BF_{10}$ = 0.11 | .028 [-.152, .205] $BF_{10}$ = 0.11 | -.064 [-.240, .116] $BF_{10}$ = 0.14 | -.220* [-.383, -.043] $BF_{10}$ = 4.15 | .546*** [.407, .660] $BF_{10}$ =7.1e5 | - |
| Fixation Tau | -.008 [-.187, .170] $BF_{10}$ = 0.13 | -.314*** [-.466, -.143] $BF_{10}$ = 89.70 | -.264** [-.423, -.090] $BF_{10}$ = 6.27 | -.232 [-.394, -.056] $BF_{10}$ = 2.13 | -.741*** [-.812, -.648] $BF_{10}$ = 4.3e11 | -.101 [-.275, .079] $BF_{10}$ = 0.27 | .120 [-.060, .292] $BF_{10}$ = 0.32 |

*Note. N* = 121. Spearman rank correlation coefficients used. Values in square brackets indicate 95% confidence intervals. Bayes factors are calculated using the approach described in van Doorn et al. (2020), which permits Bayesian inference for Spearman rank correlation coefficients.

* $p < .05$, ** $p < .01$, *** $p < .001$

**Table A8**

*Correlations between Experimental Task Performance and Questionnaire Measures in Experiment 2*

| | NEO-FFI Neuroticism | NEO-FFI Extraversion | NEO-FFI Openness | NEO-FFI Agreeableness | NEO-FFI Conscientiousness | PI-99 Good | PI-99 Safe | PI-99 Enticing | PI-99 Alive |
|---|---|---|---|---|---|---|---|---|---|
| Navon Score | -.073 [-.248, .107] $BF_{10}$ = 0.15 | -.133 [-.304, .047] $BF_{10}$ = 0.20 | -.177 [-.345, .002] $BF_{10}$ = 0.47 | -.162 [-.331, .017] $BF_{10}$ = 0.30 | .026 [-.153, .204] $BF_{10}$ = 0.12 | -.043 [-.220, .136] $BF_{10}$ = 0.11 | .011 [-.168, .189] $BF_{10}$ = 0.10 | -.137 [-.308, .043] $BF_{10}$ = 0.23 | .046 [-.133, .223] $BF_{10}$ = 0.15 |
| Memory Probe Accuracy | -.139 [-.310, .040] $BF_{10}$ = 0.39 | .054 [-.125, .231] $BF_{10}$ = 0.11 | .000 [-.178, .179] $BF_{10}$ = 0.12 | .045 [-.134, .222] $BF_{10}$ = 0.14 | .091 [-.089, .265] $BF_{10}$ = 0.18 | .093 [-.087, .267] $BF_{10}$ = 0.23 | .022 [-.157, .200] $BF_{10}$ = 0.12 | .161 [-.018, .330] $BF_{10}$ = 0.48 | -.057 [-.233, .123] $BF_{10}$ = 0.13 |
| Saccade Amplitude Mean | -.171 [-.339, .008] $BF_{10}$ = 0.35 | -.042 [-.218, .138] $BF_{10}$ = 0.11 | -.307*** [-.460, -.136] $BF_{10}$ = 10.35 | -.074 [-.249, .106] $BF_{10}$ = 0.13 | .034 [-.145, .211] $BF_{10}$ = 0.13 | -.062 [-.238, .118] $BF_{10}$ = 0.12 | -.019 [-.197, .160] $BF_{10}$ = 0.12 | -.103 [-.277, .077] $BF_{10}$ = 0.20 | .119 [-.061, .291] $BF_{10}$ = 0.37 |
| Exploratory Breadth Mean | -.132 [-.303, .048] $BF_{10}$ = 0.19 | .067 [-.113, .242] $BF_{10}$ = 0.15 | -.132 [-.304, .047] $BF_{10}$ = 0.15 | .003 [-.176, .181] $BF_{10}$ = 0.12 | .029 [-.151, .206] $BF_{10}$ = 0.11 | .013 [-.166, .191] $BF_{10}$ = 0.12 | .015 [-.164, .193] $BF_{10}$ = 0.11 | .051 [-.129, .227] $BF_{10}$ = 0.15 | -.027 [-.205, .152] $BF_{10}$ = 0.11 |
| Scan Path Length Mean | -.238** [-.399, -.062] $BF_{10}$ = 0.33 | -.076 [-.251, .104] $BF_{10}$ = 0.13 | -.262** [-.421, -.087] $BF_{10}$ = 4.31 | -.027 [-.205, .152] $BF_{10}$ = 0.11 | .107 [-.073, .280] $BF_{10}$ = 0.20 | -.033 [-.210, .147] $BF_{10}$ = 0.11 | .041 [-.138, .218] $BF_{10}$ = 0.11 | -.124 [-.296, .056] $BF_{10}$ = 0.36 | .062 [-.118, .237] $BF_{10}$ = 0.16 |
| Fixation Mu | -.061 [-.237, .118] $BF_{10}$ = 0.11 | .126 [-.053, .298] $BF_{10}$ = 0.51 | .036 [-.143, .214] $BF_{10}$ = 0.14 | .053 [-.126, .230] $BF_{10}$ = 0.12 | .003 [-.175, .182] $BF_{10}$ = 0.11 | .126 [-.054, .297] $BF_{10}$ = 0.77 | .097 [-.083, .271] $BF_{10}$ = 0.42 | .168 [-.011, .336] $BF_{10}$ = 1.40 | .005 [-.173, .184] $BF_{10}$ = 0.11 |
| Fixation Sigma | -.048 [-.224, .132] $BF_{10}$ = 0.12 | .120 [-.059, .293] $BF_{10}$ = 0.28 | .024 [-.155, .202] $BF_{10}$ = 0.12 | -.002 [-.180, .177] $BF_{10}$ = 0.11 | -.008 [-.186, .171] $BF_{10}$ = 0.12 | .122 [-.058, .294] $BF_{10}$ = 0.23 | .067 [-.113, .242] $BF_{10}$ = 0.13 | .098 [-.082, .272] $BF_{10}$ = 0.22 | 0.35 [-.145, .212] $BF_{10}$ = 0.11 |
| Fixation Tau | .123 [-.057, .295] $BF_{10}$ = 0.14 | .014 [-.164, .192] $BF_{10}$ = 0.12 | .117 [-.063, .289] $BF_{10}$ = 0.19 | -.022 [-.200, .157] $BF_{10}$ = 0.11 | -.098 [-.272, .082] $BF_{10}$ = 0.15 | -.074 [-.249, .106] $BF_{10}$ = 0.14 | -.101 [-.275, .079] $BF_{10}$ = 0.17 | .042 [-.138, .219] $BF_{10}$ = 0.13 | -.098 [-.271, .082] $BF_{10}$ = 0.18 |
| NEO-FFI Neuroticism | - | | | | | | | | |

| | | | | | | | | | |
|---|---|---|---|---|---|---|---|---|---|
| NEO-FFI Extraversion | -.214*<br>[-.378, -.037]<br>$BF_{10}$ = 2.03 | - | | | | | | | |
| NEO-FFI Openness | .222*<br>[.046, .386]<br>$BF_{10}$ = 1.34 | .135<br>[-.044, .306]<br>$BF_{10}$ = 1.10 | - | | | | | | |
| NEO-FFI Agreeableness | -.167<br>[-.335, .012]<br>$BF_{10}$ = 0.72 | .261**<br>[.087, .420]<br>$BF_{10}$ = 12.17 | 0.120<br>[-.060, .292]<br>$BF_{10}$ = 0.36 | - | | | | | |
| NEO-FFI Conscientiousness | -.424***<br>[-.560, -.266]<br>$BF_{10}$ = 5.2e5 | .294**<br>[.122, .449]<br>$BF_{10}$ = 11.55 | -.231*<br>[-.393, -.055]<br>$BF_{10}$ = 0.82 | .350***<br>[.182, .497]<br>$BF_{10}$ = 93.78 | - | | | | |
| PI-99 Good | -.388***<br>[-..530, -.225]<br>$BF_{10}$ = 2.8e3 | .484***<br>[.334, .610]<br>$BF_{10}$ = 1.0e9 | .188*<br>[.009, .354]<br>$BF_{10}$ = 1.49 | .442***<br>[.286, .575]<br>$BF_{10}$ = 1.6e7 | .350***<br>[.182, .497]<br>$BF_{10}$ = 47.38 | - | | | |
| PI-99 Safe | -.347***<br>[-.495, -.180]<br>$BF_{10}$ = 1.6e3 | .318***<br>[.148, .470]<br>$BF_{10}$ = 74.59 | .048<br>[-.131, .225]<br>$BF_{10}$ = 0.12 | .365***<br>[.200, .510]<br>$BF_{10}$ = 602.36 | .273**<br>[.100, .431]<br>$BF_{10}$ = 34.58 | .879***<br>[.831, .914]<br>$BF_{10}$ = 1.3e40 | - | | |
| PI-99 Enticing | -.190*<br>[-.357, -.012]<br>$BF_{10}$ = 0.75 | .431***<br>[.273, .566]<br>$BF_{10}$ = 1.1e3 | .333***<br>[.164, .483]<br>$BF_{10}$ = | .454***<br>[.300, .585]<br>$BF_{10}$ = 527.43 | .159<br>[-.020, .328]<br>$BF_{10}$ = 3.7e4 | .779***<br>[.697, .841]<br>$BF_{10}$ = 0.44 | .495***<br>[.348, .619]<br>$BF_{10}$ = 1.9e18 | - | |
| PI-99 Alive | -.168<br>[-.337, .011]<br>$BF_{10}$ = 0.55 | .372***<br>[.208, .517]<br>$BF_{10}$ = 267.71 | -.177<br>[-.344, .002]<br>$BF_{10}$ = 0.32 | -.043<br>[-.219, .137]<br>$BF_{10}$ = 0.11 | .309***<br>[.138, .510]<br>$BF_{10}$ = 411.51 | .365***<br>[.199, .510]<br>$BF_{10}$ = 4.6e3 | .212*<br>[.035, .376]<br>$BF_{10}$ = 2.09 | .256**<br>[.081, .415]<br>$BF_{10}$ = 11.11 | - |

*Note.* $N$ = 121. Spearman rank correlation coefficients used. Values in square brackets indicate 95% confidence intervals. Bayes factors are calculated using the approach described in van Doorn et al. (2020), which permits Bayesian inference for Spearman rank correlation coefficients.

* $p < .05$, ** $p < .01$, *** $p < .001$

## Part 2: Analysis of Datasets without Screening Applied

Descriptive statistics for the unscreened sample were similar to those for the screened sample reported in-text (see Table B1), and reliability for key experimental metrics was also comparable (see Table B2). For the full sample with a valid AOSPAN result in Experiment 1 ($N$ = 132), Cronbach's alpha was calculated at $\alpha$ = .852 (95% CI: [0.738, 0.906]) for AOSPAN Letters Correct as described in the main text.

For the unscreened sample in Experiment 1, key relationships from the screened sample mostly remained the same in the unscreened sample (see Table B3). Bayesian reanalysis of significant correlations showed that there was moderate support for an association between the Kimchi-Palmer and Navon tasks, and the Kimchi-Palmer task and AOSPAN Letters Correct. As in the screened sample, there was only anecdotal evidence for a relationship between the Kimchi-Palmer task and scan path length; although there was frequentist evidence for a correlation between the Kimchi-Palmer task and exploratory breadth, Bayes factor support was not present. However, for the Navon difference score, neither an association with scan path length nor an association with fixation tau remained when data were not screened, but a correlation with saccade amplitude which received strong Bayes factor support was observed. Finally, the frequentist relationship between memory probe score and fixation tau was replicated, but it did not receive Bayes factor support; a frequentist relationship between memory probe score and exploratory breadth which was only present in the unscreened data also did not receive Bayes factor support.

For Experiment 2, as in the screened sample, no correlations between Navon task performance and any eye movement behaviour metric were observed (see Table B4). Correlations between Openness and both saccadic amplitude and scan path length were present at a frequentist level as in the screened dataset, but the strength of evidence either for or against them was anecdotal. Conversely, as in the screened sample, there was moderate

evidence *against* the frequentist relationship between Neuroticism and scan path length (see Table B5).

**Table B1**

*Descriptive Statistics for Task Performance in Unscreened Samples for Experiments 1 and 2*

| Variable | Exp 1 | | | Exp 2 | | |
|---|---|---|---|---|---|---|
| | *n* | *M* | *SD* | *n* | *M* | *SD* |
| Kimchi-Palmer Score | 132 | 3.58 | 2.09 | - | - | - |
| Navon Task | | | | | | |
| Global Accuracy (%) | 134 | 98.20 | 2.06 | 136 | 98.90 | 1.36 |
| Local Accuracy (%) | 134 | 97.61 | 2.75 | 136 | 98.06 | 2.34 |
| Global RT (ms) | 134 | 654.59 | 178.28 | 136 | 657.98 | 185.91 |
| Local RT (ms) | 134 | 706.75 | 223.38 | 136 | 698.02 | 224.75 |
| Preference Score (ms) | 134 | 52.16 | 93.78 | 136 | 40.05 | 84.96 |
| Free Viewing Task | | | | | | |
| Memory Test (%) | 134 | 93.70 | 7.00 | 136 | 94.20 | 7.40 |
| Fixation Duration (mu) | 133 | 157.83 | 27.76 | 132 | 152.63 | 29.38 |
| Fixation Duration (sigma) | 133 | 56.58 | 16.08 | 132 | 55.04 | 13.03 |
| Fixation Duration (tau) | 133 | 149.90 | 50.49 | 132 | 147.84 | 43.44 |
| Saccadic Amplitude (°) | 133 | 4.41 | 0.84 | 132 | 4.46 | 0.79 |
| Exploratory Breadth (pixels) | 133 | 246.16 | 37.33 | 132 | 255.52 | 42.63 |
| Scan Path Length (°) | 133 | 71.24 | 18.23 | 132 | 72.63 | 20.22 |
| AOSPAN | | | | | | |
| AOSPAN Score | 133 | 46.77 | 16.91 | - | - | - |
| Letters Correct | 133 | 61.42 | 11.91 | - | - | - |
| Maths Total Errors | 133 | 3.40 | 3.84 | - | - | - |
| Maths Speed Errors | 133 | 1.09 | 1.89 | - | - | - |

| | | | | | | |
|---|---|---|---|---|---|---|
| Maths Accuracy Errors | 133 | 2.31 | 2.89 | - | - | - |
| NEO-FFI-3 | | | | | | |
| Openness | - | - | - | 136 | 43.34 | 5.86 |
| Conscientiousness | - | - | - | 136 | 42.10 | 7.91 |
| Extroversion | - | - | - | 136 | 40.43 | 6.17 |
| Agreeableness | - | - | - | 136 | 44.78 | 6.22 |
| Neuroticism | - | - | - | 136 | 37.75 | 9.13 |
| PI-99 | | | | | | |
| Good (mean) | - | - | - | 136 | 3.98 | 0.53 |
| Safe (mean) | - | - | - | 136 | 3.72 | 0.65 |
| Enticing (mean) | - | - | - | 136 | 4.34 | 0.64 |
| Alive (mean) | - | - | - | 136 | 3.57 | 0.74 |

**Table B2**

*Spearman-Brown Corrected Reliability Estimates for Selected Experimental Tasks in Unscreened Sample for Experiments 1 and 2*

| | Experiment 1 | | | Experiment 2 | | |
|---|---|---|---|---|---|---|
| | | 95% CI | | | 95% CI | |
| Task | $r_{SB}$[a] | Lower | Upper | $r_{SB}$ | Lower | Upper |
| Navon Preference Score | 0.85 | 0.77 | 0.91 | 0.82 | 0.71 | 0.89 |
| Free Viewing Task | | | | | | |
| Fixation Duration | 0.98 | 0.98 | 0.99 | 0.98 | 0.98 | 0.99 |
| Saccadic Amplitude | 0.98 | 0.97 | 0.98 | 0.98 | 0.97 | 0.98 |
| Exploratory Breadth | 0.98 | 0.97 | 0.98 | 0.98 | 0.98 | 0.99 |
| Scan Path Length | 0.96 | 0.95 | 0.97 | 0.97 | 0.97 | 0.98 |

[a] Spearman-Brown corrected reliability estimate.

*Note. N* for Navon task = 134 in Experiment 1, 136 in Experiment 2. *N* for free viewing task = 133 in Experiment 1, 132 in Experiment 2.

**Table B3**

*Correlations between Measures of Interest for Unscreened Sample in Experiment 1*

| | Kimchi-Palmer Score | Navon Score | AOSPAN Letters Correct | Memory Probe Accuracy | Saccade Amplitude Mean | Exploratory Breadth Mean | Scan Path Mean | Fixation Mu | Fixation Sigma |
|---|---|---|---|---|---|---|---|---|---|
| Navon Score | .294*** [.129, .443] $N$ = 131 $BF_{10}$ = 3.94 | - | | | | | | | |
| AOSPAN Letters Correct | .223* [.052, .380] $N$ = 130 $BF_{10}$ = 3.38 | .095 [-.077, .262] $N$ = 132 $BF_{10}$ = 0.11 | - | | | | | | |
| Memory Probe Accuracy | .007 [-.164, .177] $N$ = 132 $BF_{10}$ = 0.12 | -.034 [-.203, .137] $N$ = 133 $BF_{10}$ = 0.12 | .179* [.009, .340] $N$ = 132 $BF_{10}$ = 0.31 | - | | | | | |
| Saccade Amplitude Mean | .095 [-.078, .263] $N$ = 130 $BF_{10}$ = 0.18 | .211* [.042, .369] $N$ = 132 $BF_{10}$ = 13.59 | -.047 [-.216, .125] $N$ = 132 $BF_{10}$ = 0.11 | -.135 [-.299, .037] $N$ = 132 $BF_{10}$ = 0.24 | - | | | | |
| Exploratory Breadth Mean | .185* [.014, .347] $N$ = 130 $BF_{10}$ = 1.31 | .005 [-.166, .176] $N$ = 132 $BF_{10}$ = 0.12 | -.024 [-.194, .107] $N$ = 132 $BF_{10}$ = 0.12 | .205* [.036, .363] $N$ = 132 $BF_{10}$ = 1.03 | .465*** [.320, .589] $N$ = 133 $BF_{10}$ = 2.4e6 | - | | | |
| Scan Path Length Mean | .185* [.014, .347] $N$ = 130 $BF_{10}$ = 2.23 | .134 [-.038, .298] $N$ = 132 $BF_{10}$ = 1.72 | .040 [-.132, .209] $N$ = 132 $BF_{10}$ = 0.21 | .160 [-.011, .322] $N$ = 132 $BF_{10}$ = 0.79 | .619*** [.502, .714] $N$ = 133 $BF_{10}$ = 1.5e9 | .652*** [.541, .740] $N$ = 133 $BF_{10}$ = 5.4e14 | - | | |
| Fixation Mu | -.087 [-.256, .086] | -.003 [-.174, .168] | .034 [-.138, .204] | .070 [-.103, .238] | .201* [.032, .359] | .054 [-.118, .222] | -.076 [-.245, .095] | - | |

| | | | | | | | | | |
|---|---|---|---|---|---|---|---|---|---|
| | *N* = 130<br>BF$_{10}$ = 0.31 | *N* = 132<br>BF$_{10}$ = 0.33 | *N* = 132<br>BF$_{10}$ = 0.16 | *N* = 132<br>BF$_{10}$ = 0.21 | *N* = 133<br>BF$_{10}$ = 0.11 | *N* = 133<br>BF$_{10}$ = 0.23 | *N* = 133<br>BF$_{10}$ = 9.20 | | |
| Fixation Sigma | -.048<br>[-.219, .125]<br>*N* = 130<br>BF$_{10}$ = 0.37 | .110<br>[-.062, .275]<br>*N* = 132<br>BF$_{10}$ = 0.14 | .025<br>[-.146, .195]<br>*N* = 132<br>BF$_{10}$ = 0.11 | .012<br>[-.159, .183]<br>*N* = 132<br>BF$_{10}$ = 0.11 | .202*<br>[.033, .360]<br>*N* = 133<br>BF$_{10}$ = 0.17 | -.059<br>[-.227, .112]<br>*N* = 133<br>BF$_{10}$ = 0.31 | -.147<br>[-.310, .024]<br>*N* = 133<br>BF$_{10}$ = 1.75 | .699***<br>[.600, .777]<br>*N* = 133<br>BF$_{10}$ = 2.0e4 | - |
| Fixation Tau | -.155<br>[-.319, .017]<br>*N* = 130<br>BF$_{10}$ = 0.11 | -.126<br>[-.291, .046]<br>*N* = 132<br>BF$_{10}$ = 0.18 | -.102<br>[-.268, .070]<br>*N* = 132<br>BF$_{10}$ = 0.19 | -.336***<br>[-.479, -.175]<br>*N* = 132<br>BF$_{10}$ = 0.62 | -.020<br>[-.190, .150]<br>*N* = 133<br>BF$_{10}$ = 0.12 | -.315***<br>[-.460, -.153]<br>*N* = 133<br>BF$_{10}$ = 4.24 | -.654***<br>[-.742, -.544]<br>*N* = 133<br>BF$_{10}$ = 3.10 | -.003<br>[-.173, .167]<br>*N* = 133<br>BF$_{10}$ = 0.23 | .111<br>[-.060, .276]<br>*N* = 133<br>BF$_{10}$ = 0.19 |

*Note.* Spearman rank correlation coefficients used. Values in square brackets indicate 95% confidence intervals. Bayes factors are calculated using the approach described in van Doorn et al. (2020), which permits Bayesian inference for Spearman rank correlation coefficients.

* $p < .05$, ** $p < .01$, *** $p < .001$

**Table B4**

*Correlations between Experimental Task Performance for Unscreened Sample in Experiment 2*

| | Navon Score | Memory Probe Accuracy | Saccade Amplitude Mean | Exploratory Breadth Mean | Scan Path Mean | Fixation Mu | Fixation Sigma |
|---|---|---|---|---|---|---|---|
| Memory Probe Accuracy | .033<br>[-.136, .201]<br>*N* = 136<br>$BF_{10}$ = 0.11 | - | | | | | |
| Saccade Amplitude Mean | .103<br>[-.069, .269]<br>*N* = 132<br>$BF_{10}$ = 0.28 | .181*<br>[.010, .341]<br>*N* = 132<br>$BF_{10}$ = 0.67 | - | | | | |
| Exploratory Breadth Mean | -.030<br>[-.200, .142]<br>*N* = 132<br>$BF_{10}$ = 0.10 | .354***<br>[.195, .495]<br>*N* = 132<br>$BF_{10}$ = 113.90 | .541***<br>[.408, .652]<br>*N* = 132<br>$BF_{10}$ = 8.4e11 | - | | | |
| Scan Path Length Mean | .130<br>[-.041, .295]<br>*N* = 132<br>$BF_{10}$ =0.39 | .363***<br>[.205, .503]<br>*N* = 132<br>$BF_{10}$ = 61.07 | .736***<br>[.647, .806]<br>*N* = 132<br>$BF_{10}$ = 9.9e28 | .539***<br>[.405, .650]<br>*N* = 132<br>$BF_{10}$ = 8.6e8 | - | | |
| Fixation Mu | -.038<br>[-.207, .134]<br>*N* = 132<br>$BF_{10}$ = 0.11 | .128<br>[-.044, .292]<br>*N* = 132<br>$BF_{10}$ = 0.19 | .133<br>[-.039, .297]<br>*N* = 132<br>$BF_{10}$ = 17.00 | -.030<br>[-.200, .142]<br>*N* = 132<br>$BF_{10}$ = 0.22 | .015<br>[-.156, .186]<br>*N* = 132<br>$BF_{10}$ = 1.32 | - | |
| Fixation Sigma | .028<br>[-.143, .198]<br>*N* = 132<br>$BF_{10}$ = 0.21 | .008<br>[-.163, .179]<br>*N* = 132<br>$BF_{10}$ = 0.11 | .000<br>[-.171, .171]<br>*N* = 132<br>$BF_{10}$ = 0.42 | -.117<br>[-.282, .055]<br>*N* = 132<br>$BF_{10}$ = 0.11 | -.198*<br>[-.356, -.028]<br>*N* = 132<br>$BF_{10}$ = 0.11 | .599***<br>[.477, .698]<br>*N* = 132<br>$BF_{10}$ = 6.1e14 | - |
| Fixation Tau | -.053<br>[-.222, .119] | -.343***<br>[-.486, -.183] | -.345***<br>[-.487, -.185] | .294***<br>[-.442, -.129] | -.764***<br>[-.827, -.682] | -.101<br>[-.268, .071] | .198<br>[-.059, .278] |

| | | | | | | | |
|---|---|---|---|---|---|---|---|
| | *N* = 132<br>$BF_{10}$ = 0.11 | *N* = 132<br>$BF_{10}$ = 957.32 | *N* = 132<br>$BF_{10}$ = 0.68 | *N* = 132<br>$BF_{10}$ = 0.21 | *N* = 132<br>$BF_{10}$ = 3.3e5 | *N* = 132<br>$BF_{10}$ = 0.14 | *N* = 132<br>$BF_{10}$ = 7.46 |

*Note.* Spearman rank correlation coefficients used. Values in square brackets indicate 95% confidence intervals. Bayes factors are calculated using the approach described in van Doorn et al. (2020), which permits Bayesian inference for Spearman rank correlation coefficients.

* $p < .05$, ** $p < .01$, *** $p < .001$

**Table B5**

*Correlations between Experimental Task Performance and Questionnaire Measures for Unscreened Sample in Experiment 2*

| | NEO-FFI Neuroticism | NEO-FFI Extraversion | NEO-FFI Openness | NEO-FFI Agreeableness | NEO-FFI Conscientiousness | PI-99 Good | PI-99 Safe | PI-99 Enticing | PI-99 Alive |
|---|---|---|---|---|---|---|---|---|---|
| Navon Score | -.117<br>[-.280, .053]<br>$N$ = 136<br>$BF_{10}$ = 0.22 | -.088<br>[-.253, .082]<br>$N$ = 136<br>$BF_{10}$ = 0.14 | -.145<br>[-.306, .024]<br>$N$ = 136<br>$BF_{10}$ = 0.34 | -.108<br>[-.271, .062]<br>$N$ = 136<br>$BF_{10}$ = 0.23 | .046<br>[-.123, .213]<br>$N$ = 136<br>$BF_{10}$ = 0.12 | -.014<br>[-.182, .154]<br>$N$ = 136<br>$BF_{10}$ = 0.12 | .053<br>[-.117, .219]<br>$N$ = 136<br>$BF_{10}$ = 0.10 | -.099<br>[-.263, .070]<br>$N$ = 136<br>$BF_{10}$ = 0.12 | .034<br>[-.135, .201]<br>$N$ = 136<br>$BF_{10}$ = 0.12 |
| Memory Probe Accuracy | -.142<br>[-.304, .027]<br>$N$ = 136<br>$BF_{10}$ = 0.33 | .098<br>[-.071, .262]<br>$N$ = 136<br>$BF_{10}$ = 0.15 | .067<br>[-.103, .232]<br>$N$ = 136<br>$BF_{10}$ = 0.19 | .150<br>[ -.018, .311]<br>$N$ = 136<br>$BF_{10}$ = 0.90 | .124<br>[-.045, .287]<br>$N$ = 136<br>$BF_{10}$ = 0.30 | .137<br>[-.032, .298]<br>$N$ = 136<br>$BF_{10}$ = 0.43 | .079<br>[-.091, .244]<br>$N$ = 136<br>$BF_{10}$ = 0.17 | .194*<br>[.026, .350]<br>$N$ = 136<br>$BF_{10}$ = 0.78 | -.091<br>[-.255, .079]<br>$N$ = 136<br>$BF_{10}$ = 0.19 |
| Saccade Amplitude Mean | -.110<br>[-.276, .062]<br>$N$ = 132<br>$BF_{10}$ = 0.14 | -.035<br>[-.205, .137]<br>$N$ = 132<br>$BF_{10}$ = 0.10 | -.236**<br>[-.391, -.068]<br>$N$ = 132<br>$BF_{10}$ = 2.09 | -.026<br>[-.196, .146]<br>$N$ = 132<br>$BF_{10}$ = 0.13 | .089<br>[-.083, .256]<br>$N$ = 132<br>$BF_{10}$ = 0.14 | -.040<br>[-.210, .132]<br>$N$ = 132<br>$BF_{10}$ = 0.10 | -.003<br>[-.174, .168]<br>$N$ = 132<br>$BF_{10}$ = 0.10 | -.104<br>[-.270, .068]<br>$N$ = 132<br>$BF_{10}$ = 0.24 | .067<br>[-.105, .235]<br>$N$ = 132<br>$BF_{10}$ = 0.15 |
| Exploratory Breadth Mean | -.068<br>[-.236, .104]<br>$N$ = 132<br>$BF_{10}$ = 0.11 | .038<br>[-.134, .207]<br>$N$ = 132<br>$BF_{10}$ = 0.11 | -.080<br>[-.248, .092]<br>$N$ = 132<br>$BF_{10}$ = 0.15 | .013<br>[-.159, .183]<br>$N$ = 132<br>$BF_{10}$ = 0.11 | .052<br>[-.120, .221]<br>$N$ = 132<br>$BF_{10}$ = 0.10 | .002<br>[-.169, .172]<br>$N$ = 132<br>$BF_{10}$ = 0.11 | .003<br>[-.168, .174]<br>$N$ = 132<br>$BF_{10}$ = 0.10 | .004<br>[-.167, .175]<br>$N$ = 132<br>$BF_{10}$ = 0.10 | -.070<br>[-.238, .102]<br>$N$ = 132<br>$BF_{10}$ = 0.11 |
| Scan Path Length Mean | -.204*<br>[-.363, -.035]<br>$N$ = 132<br>$BF_{10}$ = 0.26 | -.041<br>[-.210, .131]<br>$N$ = 132<br>$BF_{10}$ = 0.10 | -.176*<br>[-.337, -.005]<br>$N$ = 132<br>$BF_{10}$ = 0.62 | .039<br>[-.133, .208]<br>$N$ = 132<br>$BF_{10}$ = 0.10 | .177*<br>[.006, .338]<br>$N$ = 132<br>$BF_{10}$ = 0.43 | .017<br>[-.155, .187]<br>$N$ = 132<br>$BF_{10}$ = 0.11 | .079<br>[-.093, .246]<br>$N$ = 132<br>$BF_{10}$ = 0.15 | -.104<br>[-.270, .068]<br>$N$ = 132<br>$BF_{10}$ = 0.29 | .042<br>[-.130, .211]<br>$N$ = 132<br>$BF_{10}$ = 0.12 |
| Fixation Mu | -.062<br>[-.230, .110]<br>$N$ = 132<br>$BF_{10}$ = 0.11 | .166<br>[-.005, .327]<br>$N$ = 132<br>$BF_{10}$ = 1.51 | .034<br>[-.138, .203]<br>$N$ = 132<br>$BF_{10}$ = 0.10 | .091<br>[-.081, .258]<br>$N$ = 132<br>$BF_{10}$ = 0.11 | .021<br>[-.150, .191]<br>$N$ = 132<br>$BF_{10}$ = 0.10 | .134<br>[-.037, .298]<br>$N$ = 132<br>$BF_{10}$ = 0.84 | .134<br>[-.037, .299]<br>$N$ = 132<br>$BF_{10}$ = 0.59 | .215*<br>[.046, .373]<br>$N$ = 132<br>$BF_{10}$ = 1.50 | -.013<br>[-.184, .158]<br>$N$ = 132<br>$BF_{10}$ = 0.11 |
| Fixation Sigma | -.048<br>[-.217, .124]<br>$N$ = 132 | .119<br>[-.053, .284]<br>$N$ = 132 | .029<br>[-.142, .199]<br>$N$ = 132 | .032<br>[-.139, .202]<br>$N$ = 132 | -.006<br>[-.177, .165]<br>$N$ = 132 | .112<br>[-.060, .278]<br>$N$ = 132 | .075<br>[-.097, .243]<br>$N$ = 132 | .159<br>[-.012, .321]<br>$N$ = 132 | .011<br>[-.161, .181]<br>$N$ = 132 |

| | | | | | | | | | |
|---|---|---|---|---|---|---|---|---|---|
| | $BF_{10}$ = 0.11 | $BF_{10}$ = 0.36 | $BF_{10}$ = 0.10 | $BF_{10}$ = 0.11 | $BF_{10}$ = 0.24 | $BF_{10}$ = 0.17 | $BF_{10}$ = 0.40 | $BF_{10}$ = 0.11 | $BF_{10}$ = 0.12 |
| Fixation Tau | .086<br>[-.086, .253]<br>*N* = 132<br>$BF_{10}$ = 0.12 | .011<br>[-.161, .181]<br>*N* = 132<br>$BF_{10}$ = 0.11 | .036<br>[-.135, .206]<br>*N* = 132<br>$BF_{10}$ = 0.11 | -.065<br>[-.234, .107]<br>*N* = 132<br>$BF_{10}$ = 0.21 | -.158<br>[-.320, .013]<br>*N* = 132<br>$BF_{10}$ = 0.53 | -.085<br>[-.252, .087]<br>*N* = 132<br>$BF_{10}$ = 0.13 | -.109<br>[-.275, .063]<br>*N* = 132<br>$BF_{10}$ = 0.15 | .043<br>[-.129, .212]<br>*N* = 132<br>$BF_{10}$ = 0.11 | -.049<br>[-.218, .123]<br>*N* = 132<br>$BF_{10}$ = 0.10 |
| NEO-FFI Neuroticism | - | | | | | | | | |
| NEO-FFI Extraversion | -.248**<br>[-.400, -.083]<br>*N* = 136<br>$BF_{10}$ = 12.25 | - | | | | | | | |
| NEO-FFI Openness | .233**<br>[.067, .386]<br>*N* = 136<br>$BF_{10}$ = 2.07 | .071<br>[-.098, .237]<br>*N* = 136<br>$BF_{10}$ = 0.33 | - | | | | | | |
| NEO-FFI Agreeableness | -.160<br>[-.319, .009]<br>*N* = 136<br>$BF_{10}$ = 0.64 | .266**<br>[.102, .416]<br>*N* = 136<br>$BF_{10}$ = 12.70 | .173*<br>[.005, .332]<br>*N* = 136<br>$BF_{10}$ = 1.26 | - | | | | | |
| NEO-FFI Conscientiousness | -.414***<br>[-.544, -.264]<br>*N* = 136<br>$BF_{10}$ = 5.6e3 | .310***<br>[.149, .454]<br>*N* = 136<br>$BF_{10}$ = 83.46 | -.189*<br>[-.346, -.022]<br>*N* = 136<br>$BF_{10}$ = 0.47 | .341***<br>[.184, .482]<br>*N* = 136<br>$BF_{10}$ = 198.42 | - | | | | |
| PI-99 Good | -.369***<br>[-.506, -.214]<br>*N* = 136<br>$BF_{10}$ = 5.2e4 | .486***<br>[.346, .605]<br>*N* = 136<br>$BF_{10}$ = 1.4e5 | .181*<br>[.013, .339]<br>*N* = 136<br>$BF_{10}$ = 1.47 | .466***<br>[.324, .589]<br>*N* = 136<br>$BF_{10}$ = 6.4e3 | .363***<br>[.208, .501]<br>*N* = 136<br>$BF_{10}$ = 214.62 | - | | | |
| PI-99 Safe | -.342***<br>[-.483, -.184]<br>*N* = 136<br>$BF_{10}$ = 1.7e3 | .349***<br>[.192, .489]<br>*N* = 136<br>$BF_{10}$ = 1.2e3 | .041<br>[-.128, .208]<br>*N* = 136<br>$BF_{10}$ = 0.11 | .384***<br>[.230, .519]<br>*N* = 136<br>$BF_{10}$ = 557.4 | .281***<br>[.119, .429]<br>*N* = 136<br>$BF_{10}$ = 45.56 | .880***<br>[.835, .913]<br>*N* = 136<br>$BF_{10}$ = 9.7e49 | - | | |
| PI-99 Enticing | -.191*<br>[-.349, -.024] | .422***<br>[.273, .551] | .329***<br>[.170, .472] | .487***<br>[.347, .605] | .148<br>[-.021, .308] | .766***<br>[.686, .828] | .514***<br>[.379, .628] | - | |

| | | | | | | | | | |
|---|---|---|---|---|---|---|---|---|---|
| | *N* = 136<br>$BF_{10}$ = 0.86 | *N* = 136<br>$BF_{10}$ = 3.0e4 | *N* = 136<br>$BF_{10}$ = 178.92 | *N* = 136<br>$BF_{10}$ = 7.3e5 | *N* = 136<br>$BF_{10}$ = 0.34 | *N* = 136<br>$BF_{10}$ = 1.4e24 | *N* = 136<br>$BF_{10}$ = 2.9e5 | | |
| PI-99 Alive | -.198*<br>[-.355, -.031]<br>*N* = 136<br>$BF_{10}$ = 1.53 | .370***<br>[.215, .507]<br>*N* = 136<br>$BF_{10}$ = 956.00 | -.203*<br>[-.359, -.036]<br>*N* = 136<br>$BF_{10}$ = 0.60 | -.070<br>[-.236, .099]<br>*N* = 136<br>$BF_{10}$ = 0.10 | .299***<br>[.137, .445]<br>*N* = 136<br>$BF_{10}$ = 1.5e3 | .345***<br>[.188, .485]<br>*N* = 136<br>$BF_{10}$ = 4.0e3 | .206*<br>[.039, .361]<br>*N* = 136<br>$BF_{10}$ = 2.86 | .203*<br>[.036, .359]<br>*N* = 136<br>$BF_{10}$ = 3.58 | - |

*Note*. *N* = 121. Spearman rank correlation coefficients used. Values in square brackets indicate 95% confidence intervals. Bayes factors are calculated using the approach described in van Doorn et al. (2020), which permits Bayesian inference for Spearman rank correlation coefficients.

* $p < .05$, ** $p < .01$, *** $p < .001$
